# Leveraging existing grid infrastructure for rapid US renewable and storage deployment

**DOI:** 10.1073/pnas.2603919123

**Published:** 2026-10-19

**Authors:** Emilia Chojkiewicz, Umed Paliwal, Duncan Callaway, Amol Phadke

**Affiliations:** ^a^Energy and Resources Group, University of California Berkeley, Berkeley, CA 94720; ^b^Goldman School of Public Policy, University of California Berkeley, Berkeley, CA 94720

**Keywords:** power systems, decarbonization, renewable energy, battery storage, energy policy

## Abstract

Rapid electricity demand growth, grid connection delays, and supply chain constraints create a serious risk of supply shortages and rising electricity costs in the next few years. This paper studies the strategy of adding colocated renewables and storage at existing power plants to take advantage of spare grid capacity. Using satellite imagery, we assess solar and wind potential near 3,890 U.S. fossil and renewable plants. By 2030, we find that existing fossil plants could host up to 597 GW of economically viable solar and wind without exceeding the existing interconnection capacity of each site. Existing renewable plants could host an additional 260 GW of firm capacity—comprising storage paired with added solar and wind capacity. These additions could accelerate clean energy deployment and enhance reliability and affordability, but realizing this potential is contingent upon regulatory reforms and coordinating the operational constraints of colocated resources.

## Significance

Meeting rapidly rising U.S. electricity demand while maintaining reliability and affordability is increasingly constrained by delays and high costs of new grid infrastructure. Although policy has focused on building new generation, transmission, or gas-fired capacity, existing power plants’ grid connections are often significantly underutilized. We find that leveraging this spare capacity by allowing new clean generation and storage to share existing interconnection rights offers a fast, cost-efficient pathway with substantial opportunity: 597 GW at existing fossil plants and 260 GW at existing renewable plants by 2030. Yet surplus interconnection remains a niche practice, hindered by fragmentation and regulatory uncertainty. Targeted reforms to interconnection policies could unlock this underused strategy as a central tool for near-term reliability and clean energy deployment.

Power demand is surging, driven by a confluence of factors including electrification, reshoring of manufacturing, and data centers for AI. While there is significant uncertainty in the quantity, location, and timing of this load growth, most estimates project a 15 to 25% increase in peak power demand by 2030 (100 to 130 GW), with some regions of the United States (US) potentially experiencing a near doubling over the same period (1–3). However, currently planned capacity additions are insufficient to reliably meet projected peak demand (3), with fears of supply shortages reflected in heightened capacity prices; for example, in a recent capacity auction for 2026/27 delivery in the PJM region, prices spiked to the cap of $329.17/MW-day for the entire footprint, up from $28.92/MW-day for the 2024/25 delivery year (4). Although this signals an urgent need for large-scale investment into grid infrastructure, efforts to bring new generation online are severely constrained by several bottlenecks in the interconnection process. New projects face median wait times of 5 to 6 y along with escalating network upgrade costs that hinder timely deployment (5). Various supply chain constraints further exacerbate the predicament: transformer lead times average 2 to 4 y (6) while the natural gas turbine industry has confirmed turbine lead times of 4 to 6 y and relatively higher capital costs (7). The costs of new gas plants have also escalated: whereas projects slated for completion in 2026/27 were reported at $1,116 to 1,427/kW, recent CCGT projects are routinely reporting costs of $2,000/kW or more (8). Together, these challenges underscore the growing risk that the pace of new resource deployment will lag behind accelerating demand growth, threatening U.S. economic competitiveness, system reliability, and affordability in the near term.

One strategy to accelerate resource deployment is to improve the utilization of existing grid infrastructure by integrating new generation and/or storage at the point of interconnection (POI) of existing power plants, as shown in Fig. 1. This approach—also referred to globally as grid connection sharing, repowering, cable pooling, or hybridization—is generally enabled in the United States through a process known as surplus interconnection service. The Federal Energy Regulatory Commission (FERC) defines surplus interconnection service as any unused portion of interconnection service established in a Large Generator Interconnection Agreement (9). In this paper, we use the term “surplus” to refer to the temporal opportunity to deliver additional energy (MWh) at a POI without exceeding the POI’s existing “interconnection capacity,” which we assume is equal to the capacity of the existing plant. Therefore, surplus interconnection service does not require network upgrades associated with increasing maximum power injections and can avoid the corresponding studies, providing a faster pathway for deploying new resources compared with the congested and time-intensive conventional interconnection process (9–11). Such projects are beginning to see real-world implementation, though on a limited scale (see the *SI Appendix*, *Supplementary Text* for case studies).

**Fig. 1.**
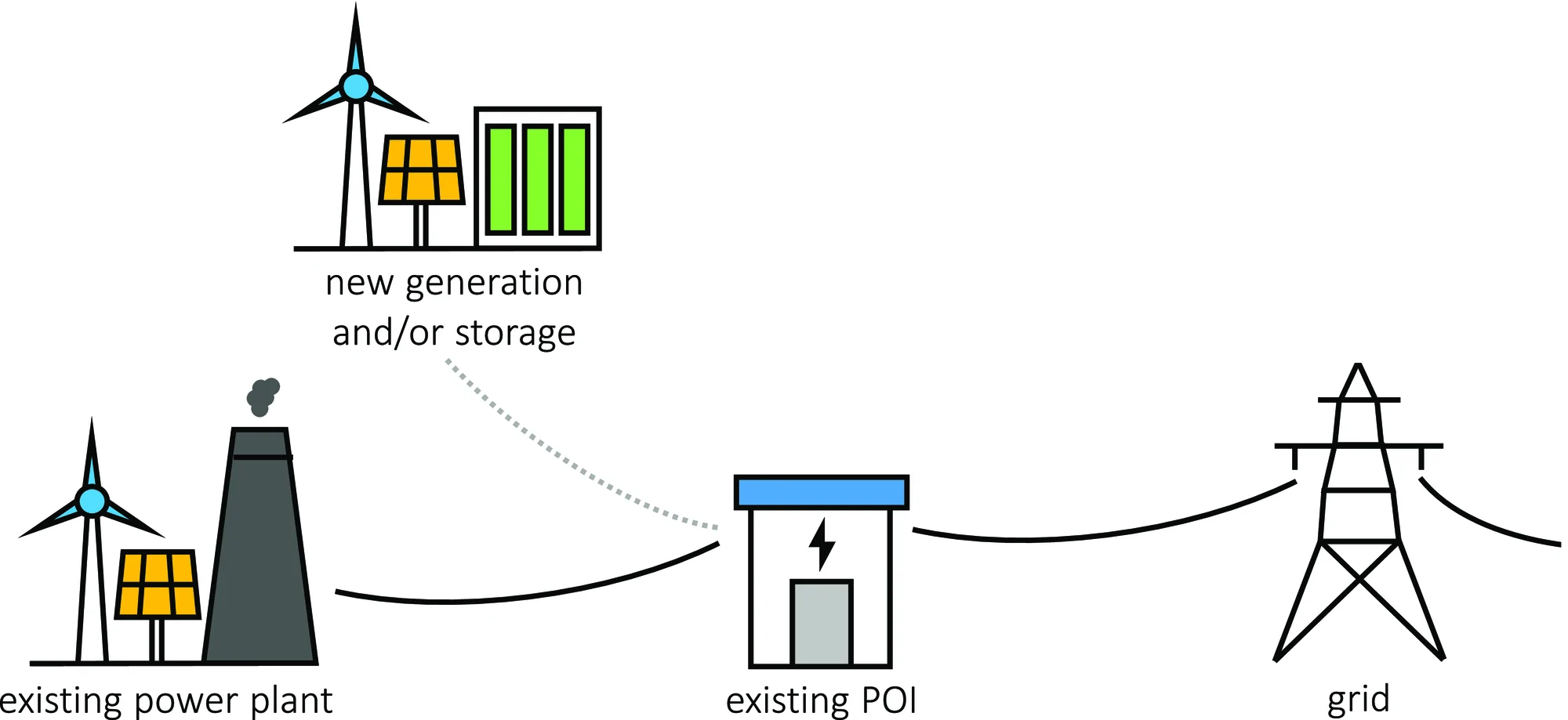
A schematic of surplus interconnection service. New generation and/or storage can share the existing POI with existing power plants through the surplus interconnection service.

This surplus interconnection opportunity has two primary drivers. First, the grid connection and associated transmission infrastructure of existing power plants is not fully utilized. For example, the United States maintains approximately 1,000 peaker plants which operate only a few percent of total annual hours to help supply periods of high residual demand; these peaker plants are typically natural gas-fired combustion turbines (CTs) as well as oil and gas steam turbines (STs) with lower efficiencies compared to baseload thermal generation (*SI Appendix*, Fig. S2) (12). Furthermore, existing utility-scale solar and wind farms in the United States operate with an average fleetwide capacity factor of 24% and 34%, respectively (*SI Appendix*, Fig. S3) (13, 14). This signals a significant underutilization of existing POIs, to which the addition of new generation and/or storage capacity could increase the electricity supply to the system at a lower cost and with faster project deployment than new generation capacity that requires a new POI. The second surplus interconnection driver is that utility-scale solar photovoltaic (PV), onshore wind, and battery storage are increasingly the lowest-cost sources of new electricity generation across much of the United States (*SI Appendix*, Fig. S4). These resources have experienced dramatic cost reductions in recent years resulting from improved manufacturing processes and the scaling of global supply chains (15), remaining cost-competitive in many locations even in the absence of the expiring tax credits from the Inflation Reduction Act (IRA) (16, 17). As a result, solar, wind, and storage account for more than 90% of new U.S. electrical generating capacity additions in 2025 (18), a trend expected to continue in 2026 (19). Furthermore, the markets for solar PV panels along with utility-scale battery storage are not experiencing the same major multiyear supply chain constraints associated with new natural gas plants (7, 8). In this context, surplus interconnection service offers a pathway to rapidly integrate energy supply from these low-cost resources at existing POIs, which in turn contains wholesale electricity prices as demand grows—advancing energy affordability.

Although the concept of interconnection sharing has been discussed for years, it was originally perceived as a limited case-by-case opportunity that required the highest RE resource quality (20–22). However, intensifying demand growth, bottlenecks in the conventional interconnection queue, and the widespread availability of low-cost renewables and storage warrant a reexamination of the opportunity (11). Recent work by RMI identified 250 GW of potential solar, wind, and storage could be integrated at the POIs of existing thermal power plants, concentrated primarily in the Midwest, Mid-Atlantic, and Southeast (23). RMI’s analysis, however, relies on IRA incentives and does not assess surplus interconnection opportunities at existing renewable plants. We build on this work by evaluating the economic and technical viability of adding new renewables and/or storage at existing power plants across the contiguous U.S., focusing on two principal classes of opportunity: existing fossil plants, where the primary value lies in rapidly integrating new lower-cost generation resources, and previously unstudied existing renewable plants, where additional renewables with battery storage can increase the supply of dispatchable capacity.

## Extensive Land Availability and Renewable Energy Potential Near Existing Power Plants

To quantify the potential for leveraging the POI of existing power plants, we first estimate the total resource potential of solar and wind in close proximity to existing power plants. While previous studies assessed the renewable potential over a large radius, i.e., 45 km (23), in order to robustly make the case there is sufficient local renewable resource that can be developed cost-effectively, we use a considerably tighter i.e., 20 × 20 km (12 × 12 mile) square buffer zone with the existing power plant at the center. This is reflective of commercial activity in the United States today (see the *SI Appendix*, *Supplementary Text* for case studies and *SI Appendix*, Tables S1 and S2). Within each buffer zone, we assess the renewable resource potential of each 30 × 30 m (100 × 100 ft) parcel of land through high-resolution satellite imagery. We replicate criteria that renewable project developers typically use to select suitable land for project deployment, incorporating over 50 exclusions grouped into two main categories: physical site characteristics (i.e., land cover, slope gradients, elevation constraints) and land use (i.e., environmental protection status, development restrictions) (for more details, see *Methods*; for the full list of exclusions, see the *SI Appendix*, *Supplementary Text*). We focus on two principal classes of opportunity: existing fossil fuel power plants, which are increasingly the most expensive sources of electricity generation, as well as existing renewable plants, which are likely underutilized due to the intermittent nature of solar and wind energy. Other electricity generation technologies are excluded based on their operating characteristics and/or limited contribution to the U.S. generation mix, yet have the potential to provide additional surplus potential. We consider plants above 10 MW in capacity, as smaller units are often located in industrial facilities and do not serve bulk grid demand.

We find that the total maximum local solar and wind potential within the 20 × 20 km buffer zone near the 1,403 existing fossil power plants in the contiguous U.S. is 15,375 GW, and the potential at the 2,487 existing renewable power plants is 26,928 GW. While these estimates represent a theoretical maximum and are unlikely to be fully realized, they demonstrate that renewable resources are widely available in close proximity to existing power plants. Nearly 90% of existing fossil and 76% of existing renewable plants have solar and wind potential that exceeds their interconnection capacity by more than five times. We also note that solar and wind need not be treated as mutually exclusive land uses: because wind turbines occupy only a small fraction of a project’s land area, solar arrays can be sited between turbines.

Fig. 2 shows the land use screening process with the resulting renewable energy potential assessment; for information on how to access the data dashboard with the full results, see *Data, Materials, and Software Availability*.

**Fig. 2.**
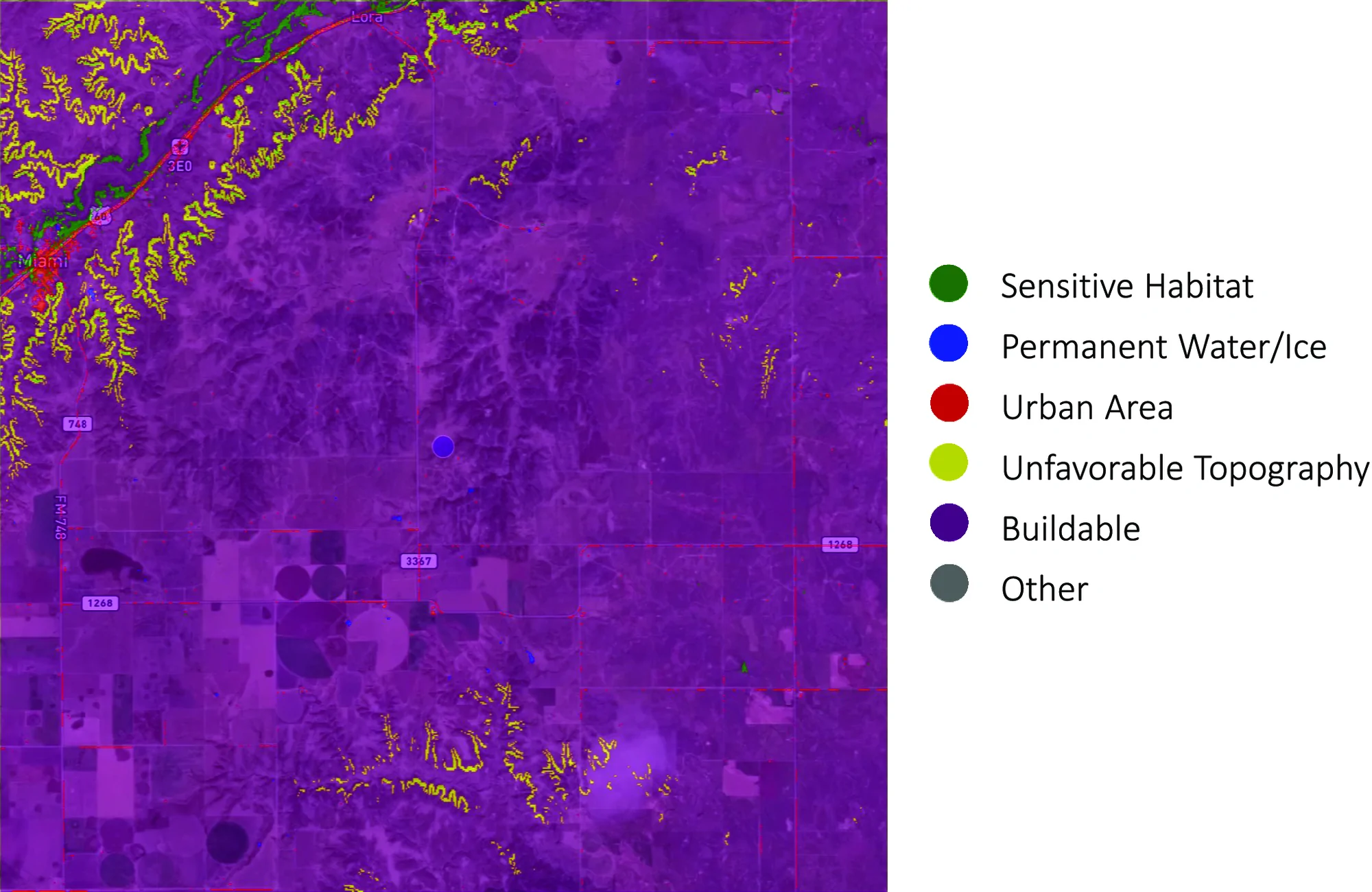
Exemplary land use-land cover screening for the Miami Wind Energy Center. Based on exclusion criteria of physical site characteristics, protected areas, infrastructure density, and regulatory restrictions, over 95% of land in the vicinity of this 289 MW wind farm in Texas has potential for renewable energy project development. We find that this 12 × 12 mile buffer zone is capable of hosting up to 372 MW of solar and 81 MW of wind. For information on how to access the data dashboard with results for all plants, see *Data, Materials, and Software Availability*.

## Surplus at Existing Fossil Plants Offers Up to 597 GW of RE Integration Potential by 2030

In this era of rapid load growth, delays in deploying new low-cost generation risk increasing electricity costs and emissions. By way of example, states with the fastest load growth in 2024—e.g., Virginia and Texas—also saw the largest increases in gas generation (24), implying more frequent reliance on expensive, carbon-intensive generation units to meet marginal demand. Surplus interconnection service at existing fossil plants offers a near-term opportunity to increase deployment of low-cost solar and wind, yet its economic viability depends on a simple comparison: the variable cost of each incumbent fossil-fired generation unit vs. the projected levelized cost of energy (LCOE) of new solar or wind capacity at the same POI. This comparison, widely used in industry assessments of renewable competitiveness (16, 17), captures the tradeoff between investing in new renewable generation vs. operating existing fossil units during hours when renewable output would be available.

This comparison is viewed in Fig. 3, where we plot the variable cost (the sum of the fuel cost and variable operation and maintenance cost) of all existing fossil-fired generation units in the United States against the projected LCOE of solar and wind at the same POI, from 2024 to 2030 (see *Methods* for details). We define “crossover locations” as existing fossil power plant sites where the projected local LCOE of solar or wind is lower than the fossil units’ variable cost (corresponding to the top left area of each subplot, above the dashed orange line). At crossover locations, renewable generation should be prioritized when available subject to operational constraints of the incumbent generator such as ramping limits, minimum output requirements, or capacity obligations. As of 2024, 60% and 53% of the 4,643 fossil units in the United States are located at crossover locations for solar and wind, respectively, corresponding to a respective 275 GW and 250 GW (40% and 36%) of fossil-fired capacity. The generation technologies at crossover locations include nearly all gas combustion turbines and oil steam turbines, roughly half of combined-cycle gas units, and a subset of coal plants that remain economically competitive in the absence of a carbon price. Continued declines in renewable costs are projected to expand the set of crossover locations to include most remaining fossil units by 2030 (*SI Appendix*, Fig. S5), representing an upper bound on the long-run economically viable potential for renewable deployment through surplus interconnection service.

**Fig. 3.**
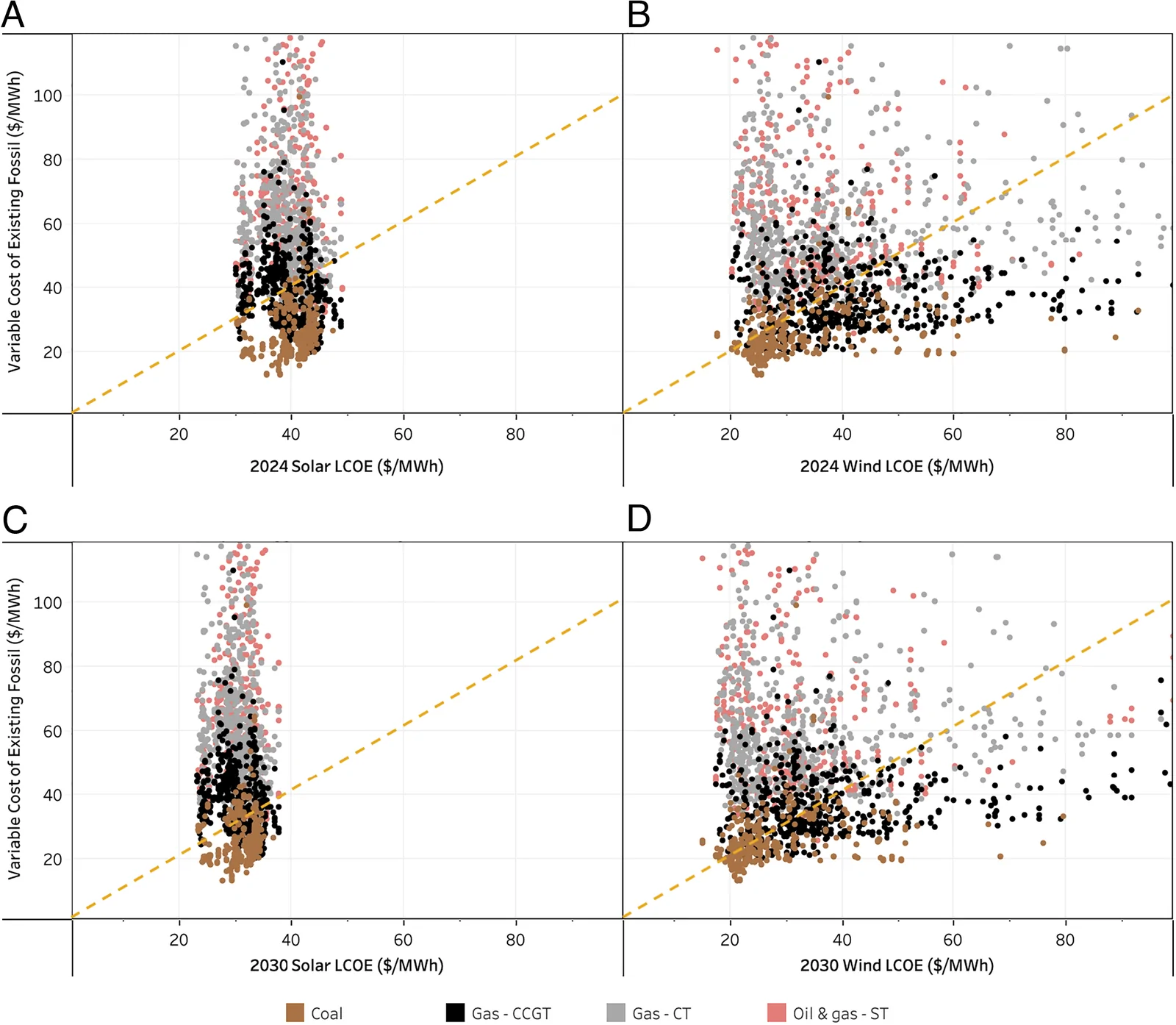
Solar and wind crossover locations encompass 53 to 60% of existing fossil units in the United States as of 2024. Local solar and wind LCOE compared with the variable cost (fuel plus variable O&M) of existing fossil-fired generating units in 2024 (*A* and *B*) and 2030 (*C* and *D*). Crossover locations are existing fossil power plant sites where the projected local renewable LCOE is lower than the fossil units’ variable cost, corresponding to the region above the dashed orange line. Each point represents a fossil-fired generating unit, colored by the unit’s fuel and technology.

The near-term opportunity amid a high load growth environment—in which, absent new surplus resources, existing fossil generators would be dispatched to serve incremental demand—can be assessed by applying variable cost cutoffs to existing fossil units. For each cutoff, we assume that fossil units with variable costs below the threshold would be economically viable to service load growth, and quantify the amount of fossil capacity at crossover locations among these units. The opportunity remains substantial: applying a variable cost cutoff of $45/MWh retains 99 GW of fossil capacity at solar crossover locations as of 2024 (*SI Appendix*, Fig. S6; results for wind are presented in *SI Appendix*). As a robustness check, we apply a second cutoff based on local solar LCOE to identify the lowest-cost development opportunities. As of 2024, 270 GW of fossil capacity is located at solar crossover locations with a local solar LCOE below $40/MWh, indicating competitive sites for new solar development (*SI Appendix*, Fig. S7). Together, these two cutoff approaches provide complementary lower-bound estimates of the near-term opportunity for renewable deployment through surplus interconnection service.

We next optimize the capacity of solar and wind that is not only economically viable, but also technically feasible for integration at each existing fossil plant. To do so, we first refine the total solar and wind potential by applying regulatory restrictions (i.e., local ordinances which prohibit wind development) and using infrastructure density as a heuristic indicator of cost-effective developable land (specifically, excluding solar and wind potential where built infrastructure exceeds 30% of the land area in the buffer zone). Then, we take each site’s local hourly solar and wind profiles over a full year and maximize the total annual renewable generation, subject to constraints including an interconnection capacity limit (to ensure that delivered renewable generation does not exceed the capacity of the existing POI, which is conservatively assumed to equal the existing plant’s nameplate capacity as reported in EIA Form 860) (25). To ensure that integrating solar and wind projects near existing fossil power plants would not compromise on quality nor cost compared to integrating renewables at new POIs, we validate the simulated generation against the performance of recently deployed renewable projects; while solar resources are generally commensurate within the 20 × 20 km buffer zone, we exclude all wind sites with a capacity factor below 30%, reflecting the minimum capacity factor observed in recently deployed projects (14). For full modeling details and assumptions, see *Methods*.

As seen in Fig. 4*A*, the solar and wind potential that is simultaneously technically feasible and economically viable comprises 412 GW of so-called “actionable” renewable potential near the interconnection of existing fossil power plants as of 2024. This capacity increases to 507 GW by 2027 and 597 GW by 2030 as the costs of new renewables are projected to decline (see *Methods* for details). Fig. 4*B* shows the breakdown of actionable renewable potential by Independent System Operator (ISO), which highlights over 200 GW of opportunity in PJM and MISO by 2030, followed by SPP, ERCOT, and ISONE. Given the impending expiry of IRA incentives in 2026, these results include neither the production nor the investment tax credits; however, we replicate the economic viability analysis incorporating IRA incentives, finding that the tax credits would increase the actionable renewable potential by 64%, to 977 GW by 2030 (*SI Appendix*, Figs. S8 and S9). It is important to note, however, that these estimates of the actionable renewable potential represent an upper bound because they do not account for existing power plants’ market obligations (e.g., capacity and ancillary services) or operational constraints that may limit surplus resources’ injections through existing POIs (e.g., ramp rate limits), although many of these constraints can be mitigated through system-level coordination, renewable forecasting, and colocated battery storage.

**Fig. 4.**
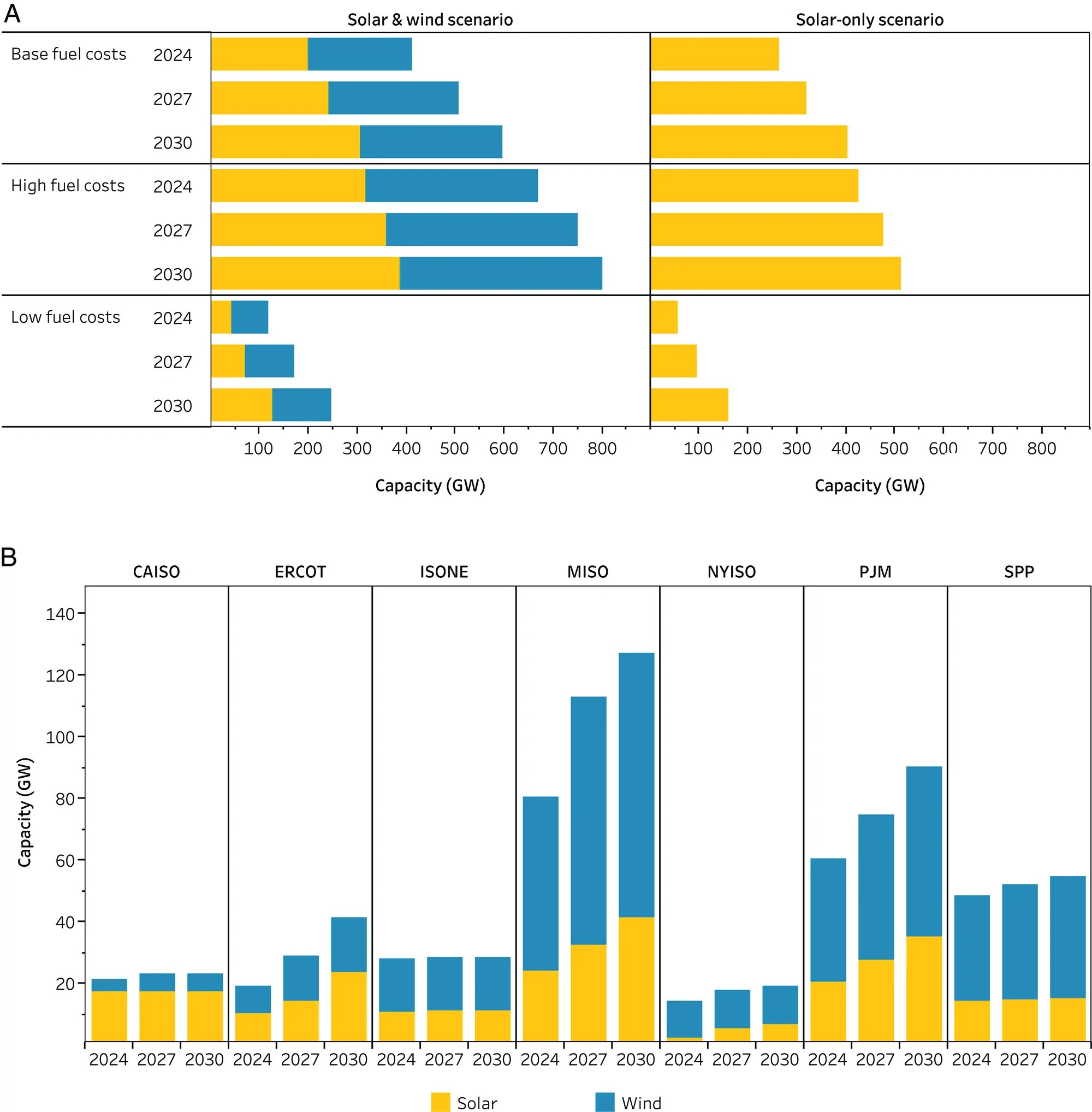
Actionable renewable integration potential at existing fossil interconnection. As of 2024, 412 GW of solar and wind is simultaneously technically feasible and economically viable to be added to the interconnection of existing fossil power plants, which increases to 507 GW by 2027 and 597 GW by 2030 as the costs of new renewables decline. The nationwide actionable potential varies by scenario (solar & wind, or solar-only), fuel costs (base, high, low), and year (2024, 2027, 2030) (*A*). The actionable potential of the solar & wind scenario with base fuel costs by year (2024, 2027, 2030) in each ISO, highlighting over 200 GW of opportunity in PJM and MISO by 2030 (*B*).

In order to assess the robustness of our findings to key input variables, we conduct two sensitivity analyses. First, because fuel prices are a critical determinant of crossover locations, yet future fuel prices are uncertain, we analyze two cases of low and high fuel prices: one SD below and above the state’s mean, relative to the base case. Fig. 4*A* shows that high fuel costs amplify the surplus opportunity by 25%, to over 800 GW by 2030; however, low fuel costs reduce the actionable renewable potential by nearly 60%, to approximately 250 GW of solar and wind by 2030, driven primarily by more competitive natural-gas fired generation. Second, considering that recent US deployment trends show declining onshore wind additions as solar additions have surged (18, 19)—reflective of solar’s relatively lower complexity associated with siting, permitting, and installation logistics—we conduct a solar-only scenario as well. We find that 404 GW of solar PV could be integrated at existing interconnection by 2030 with the base fuel costs, 513 GW of solar PV with high fuel costs and 161 GW with low fuel costs (Fig. 4*A*). An overview of the methodology and the refinement of the total potential to the actionable potential can be viewed in *SI Appendix*, Fig. S10.

There are two primary benefits of utilizing surplus interconnection at existing fossil power plants: 1) new generation resources can be deployed faster and more cost-effectively than through the congested conventional interconnection process, 2) these new resources would be dispatched before fossil resources, reducing wholesale electricity prices relative to a counterfactual in which fossil generation meets incremental load growth. One important limitation, however, is that while battery storage can likewise be integrated at these POIs—along with additional solar and wind for charging, reducing renewable curtailment resulting from export constraints—its incremental system-level value may be limited. Because the POI is already anchored by a dispatchable resource, battery storage offers relatively little additional reliability or capacity value beyond operational flexibility. Consequently, in order to unlock the potential of surplus interconnection service to supply additional firm capacity, attention must shift to existing renewable power plants.

## Surplus at Existing Renewable Plants Offers 260 GW of Firm Capacity by 2030

At existing renewable power plants, battery storage can firm the output of intermittent renewable resources; additional solar, wind, and battery storage can increase dispatchability up to the capacity of the existing POI. Because solar and wind are zero-marginal-cost resources, the crossover analysis used for existing fossil plants is not applicable. Instead, the value of surplus interconnection lies in increasing the resource dispatchability and utilization of the existing POI, supporting grid reliability. Similar to the technical feasibility analysis performed for fossil power plants, we take each site’s local hourly solar and wind profiles over a full year and maximize the total annual renewable generation (for more details, see *Methods*). We determine the optimal configuration of solar and wind at each site while fixing the rated capacity of the battery storage system as the installed capacity of the existing renewable plant with 6-h of storage duration. We include the 19 GW of renewable plants with 30 GWh of existing hybrid battery storage as of 2024, assuming their storage capacity is expanded to 6-h duration. Throughout the study, we use the term “firm capacity” to refer to dispatchable capacity capable of providing reliability services comparable to conventional peaking resources. Across the United States, 6-h battery storage systems demonstrate high capacity value in Effective Load Carrying Capability (ELCC) studies, which quantify a resource’s contribution to system reliability during peak demand. For instance, in PJM, a 6-h storage system achieves an ELCC of 58%, comparable to a gas combustion turbine at 60% (26); nationally, ELCC values for 6-h storage systems range from 60 to 80% of rated capacity, offering a cost-effective and practical benchmark for our analysis (27–29).

We find that adding 6-h battery storage to existing renewable power plants enables the addition of 324 GW of solar PV and 128 GW of wind to the same POI (Fig. 5*A*). This yields an additional 260 GW of firm capacity nationwide, corresponding to about 1,530 GWh of new battery storage capacity—a 50-fold increase relative to the 30 GWh currently paired with renewable generators on the U.S. grid. The growing value of firm capacity is reflected in recent capacity market outcomes across the United States. In PJM, clearing prices reached the cap of $329.17/MW-day in the latest auction for the 2026/27 delivery year, compared to $28.92/MW-day for 2024/25. Similarly, ISO-NE’s most recent Forward Capacity Auction (FCA 18) cleared at $3.58/kW-month, a 40% increase over the previous year’s results, while MISO’s summer 2025 capacity prices surged to $666.5/MW-day, up from $30/MW-day the previous year (4, 30, 31). These elevated prices signal tightening reserve margins and growing reliability risks. Without timely interventions, these price increases could lead to rising consumer bills and potential supply shortfalls (32, 33). Integrating renewables and battery storage at existing POIs offers a rapid and modular strategy to mitigate these risks while simultaneously reducing the need for conventional firm capacity like new gas turbines and alleviating pressure on capacity markets.

**Fig. 5.**
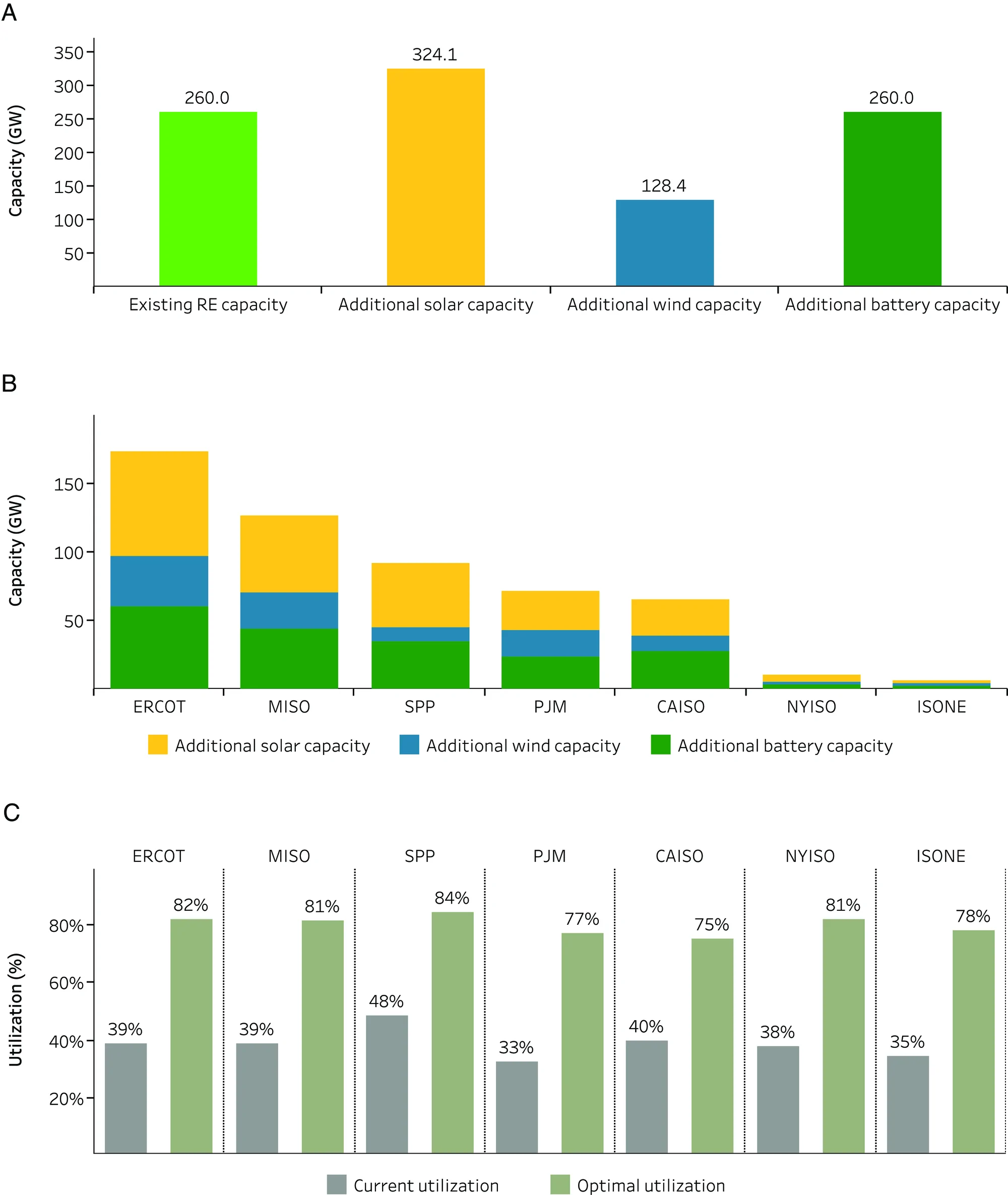
Actionable renewable integration potential at existing renewable interconnection. Adding 6-h battery storage, 324 GW of solar PV, and 128 GW of wind to existing renewable power plants enables 260 GW of additional firm capacity nationwide (*A*). The largest opportunity lies in ERCOT followed by MISO and SPP, as seen in the breakdown of actionable renewable integration by ISO (*B*). The utilization of the existing renewable plants’ POI improves by 35 to 44 percentage points across major ISOs through the addition of new renewables and 6-h storage (*C*).

The integration of battery storage and additional renewables at existing renewable plants substantially improves the utilization of the existing POI. For existing solar assets, we find that adding 108 GW of 6-h battery storage (and 101 GW of solar and 83 GW of wind) has the potential to more than double the utilization of existing renewable POIs (from 30 to 71%); meanwhile, for existing wind assets, adding 152 GW of 6-h battery storage (and 222 GW of solar and 45 GW of wind) can increase utilization rates from 47 to 83%. The regional distribution in Fig. 5*B* shows that the greatest opportunity lies in ERCOT and MISO, which can respectively add over 60 GW and nearly 44 GW of firm capacity. In both regions, this is driven by the large installed capacities of existing renewables. In addition, as seen in Fig. 5*C*, in each region the utilization of renewable interconnection improves by 35 to 44 percentage points with new resource integration through surplus interconnection service, reducing the need for new interconnection and transmission infrastructure. The largest improvement in interconnection utilization with the addition of battery storage is in PJM, from 33 to 77%. These values should be interpreted as an upper bound, constrained by existing renewable deployment, the existing plants’ deliverability, curtailment induced by grid operators, and current battery manufacturing supply chains.

Adding new renewables and battery storage to existing renewable power plants has significant potential to address much of the projected demand growth in the coming 4 to 6 y. For example, the high actionable potential at existing renewable sites particularly in ERCOT, SPP, and MISO (Fig. 5) exceeds the projected peak demand growth (53, 24, and 16 GW by 2030, respectively) (2). As some regions consider the prioritization of gas plants—with 4 to 6 y lead times—in the interconnection queue, integrating renewables and battery storage at existing interconnection could provide a much faster path to capacity buildout. This is especially pertinent considering the high uncertainty in the magnitude, location, and timing of the load growth. Whereas gas plants face a correspondingly high stranded asset risk if demand fails to materialize, renewables and batteries offer a modular option, where deployment can be staged. However, the extent to which leveraging surplus capacity at existing renewable plants can help meet peak demand growth largely depends on the level of interconnection service (i.e., deliverability) of these existing renewable plants, the ability to streamline deliverability studies and capacity accreditation for surplus resources, and the extent to which new loads can be strategically sited near plants with available surplus resources.

Utilizing existing interconnection has the added benefit of avoiding much of the costs of interconnection, including network upgrades. These costs have grown significantly in recent years: completed project costs reported in 2019–2023 averaged $81/kW but reached as high as $250/kW in some regions (2022 US$) (34). While these costs are initially incurred by project developers, they are ultimately reflected in consumer prices. Reducing the costs associated with interconnection increases the likelihood that the project will be developed, considering that these network upgrade costs have increasingly been cost-prohibitive in recent years. We estimate that leveraging surplus interconnection could save up to $51 billion at existing fossil sites and up to $109 billion at existing renewable sites in avoided interconnection costs. However, we do not consider additional benefits arising from the colocation of solar, wind, and batteries as well as the reduced need for investments in bulk transmission. In addition, some expenditure on protection equipment or other infrastructure would still be required, which is not accounted for here.

## Discussion

Leveraging existing infrastructure through surplus interconnection offers a scalable cost-effective pathway to accelerate new resource integration in the United States and globally, especially given surging electricity demand, bottlenecks in the standard interconnection process, and long lead times for new gas generation. With hundreds of GW of opportunity distributed across the continental U.S., the strategy can deliver significant cost and time savings, reducing the risks of supply shortages and enhancing grid reliability (11). Renewable developers benefit from faster project timelines and lower infrastructure costs due to avoided expenditure on interconnection equipment (10). For fossil sites, surplus interconnection can displace thermal generation with lower-cost solar and wind, reducing local air pollution while sustaining tax revenues and jobs as fossil assets retire (10). Quantifying the emissions reductions from leveraging surplus resources at crossover locations requires system dispatch modeling beyond the scope of this study. Nevertheless, these locations represent economically attractive opportunities for renewable deployment that could displace higher-cost, higher-emitting fossil generation while exerting a downward pressure on wholesale electricity prices. However, realizing this potential requires careful site-specific evaluation of technical feasibility, existing grid obligations, and community acceptance, particularly in energy communities.

Although FERC Order 845 paves the way for capitalizing on surplus interconnection service, implementation varies substantially across transmission providers and ISOs. Realizing the full technical potential identified in this study requires additional policy support to make it a mainstream strategy (9). We recommend FERC standardize the best practices, mandate consistent study timelines, and facilitate the development of pro forma agreements for sharing interconnection rights (11, 23). Further, new surplus resources at renewable sites may not qualify for capacity credits since deliverability is typically assessed separately from surplus interconnection studies (11). This limits new surplus resources to “energy-only” status despite their contributions to resource adequacy, peak load reduction, and deferred/avoided network upgrades; these benefits should be properly reflected in system planning, preventing costly overbuilding (10). Further, because surplus interconnection service is tied to the original generator’s interconnection agreement, regulators and operators should define expedited generator replacement provisions for transferring interconnection rights if the original plant retires (11). Additionally, improving transparency through public databases can help facilitate the identification of surplus opportunities, especially at generators with low utilization. Meanwhile, state policymakers should mandate the consideration of surplus resources in integrated resource planning, as was proposed but not ultimately passed in California (35), as well as providing financial incentives and aligning state permitting rules (10).

While the regulatory framework for surplus interconnection service is technology neutral, several technical questions remain, particularly around coordination and control of colocated resources. Key considerations include the designation of operational control responsibilities, simultaneous injection limits, enforcement of interconnection agreements, fault handling and ride-through, coupling of inverter based resources with conventional synchronous resources, and availability for grid services such as frequency regulation, inertia, and voltage and reactive power (11). These are crucial in an increasingly inverter-based grid, yet can also be addressed through the incorporation of grid-forming inverters. For renewable surplus, compatibility between the power electronics of the existing and newly installed resources (inverters, battery management software, etc.) is critical. These requirements may necessitate additional protection schemes and/or control systems but can be eased through standardized contractual agreements for sharing interconnection rights and delineating responsibilities between the original and added generators. Of the surplus projects deployed to date (see the *SI Appendix*, *Supplementary Text* for case studies), the operator of the new and existing plant is often the same, which can reduce information asymmetries and streamline operations. Whether this reflects a practical preference or broader market trend remains unclear, but limiting surplus interconnection opportunities to incumbent operators could reduce participation by independent developers and the competitive discovery of alternative, potentially more efficient uses of interconnection capacity. Accordingly, Order 845 grants the original interconnection customer (or its affiliate) first priority to develop surplus interconnection projects while requiring unused opportunities be offered to other prospective interconnection customers through an open and transparent solicitation process (9).

An increasingly decarbonized, reliable power system requires a diverse generation portfolio, varying by region. Solar, onshore wind, and batteries account for the majority of both new resource additions and capacity in interconnection queues in the United States today (5, 36), and as shown here, much of this portfolio could be developed via surplus interconnection. Methodologically, we use an industry-accepted approach to estimate variable costs, but actual fuel costs are considered to be highly sensitive confidential information and may vary widely by contract type, indexation, supplier, deliverability, ownership, and location (17). Accurate fuel cost data are critical for determining the economic viability of adding solar, wind, or storage at existing fossil plants, so generation owners should evaluate surplus potential or consider alternative strategies, such as auctioning valuable interconnection rights. Comparisons between fossil units’ variable costs and renewable LCOE should also consider finer temporal resolution, projected load growth, and assess electricity prices. Future work should incorporate network constraints and system-level evaluation to assess how surplus resources can support rising load growth while accounting for potential congestion and transmission bottlenecks. Ongoing work addresses these refinements through power flow modeling in real-world systems.

## Methods

### Land Use Screening.

To assess the solar and wind potential near existing fossil and renewable power plants, we conduct a geospatial analysis integrating multiple datasets, drawing on previous analyses (37–39) for methods and exclusion criteria. For each plant, we define a 20 × 20 km (12 × 12 mile) square buffer zone with the existing power plant at the center, representing the area within which new solar or wind projects could feasibly connect to existing interconnection infrastructure. Within each buffer, we evaluate the suitability of every 30 × 30 m parcel using a binary land-use screen that approximates the siting criteria used by renewable project developers. Parcels are excluded based on physical site characteristics (e.g., land cover, slope >10°, elevation >4,000 m) and land-use restrictions (e.g., protected areas, parks, military lands, and local development restrictions) (40–42). We additionally incorporate county- and municipality-level renewable energy ordinances (43, 44). A site’s developable area is calculated as the sum of all nonexcluded parcels within its buffer zone. The complete set of exclusion criteria is provided in *SI Appendix*.

### Solar and Wind Generation.

For the remaining suitable areas within each buffer zone, we estimate potential solar and wind energy capacities using standard conversion factors: 50 MW per square kilometer for solar PV and 6 MW per square kilometer for wind (39). These factors reflect typical utility-scale installation densities and provide an estimate of the maximum buildable renewable capacity near each existing power plant. Then, to obtain the hourly solar and wind generation profiles at each existing plant location, we use high-resolution meteorological data for the 2019 weather year and a custom software tool developed for the study that integrates established models with detailed system specifications. The 2019 weather year was selected as a representative meteorological year, consistent with other studies (45).

For solar, we utilize the ERA5 dataset from the European Centre for Medium-Range Weather Forecasts (ECMWF), providing hourly data on solar irradiance, air temperature, pressure, and wind speed at approximately 31 km resolution (46). For the simulation, we calculated three key solar irradiance measurements (Global Horizontal, Direct Normal, and Diffuse Horizontal) to model solar power generation. Using these measurements, we simulated the hourly output of a 1 MW solar farm equipped with single-axis tracking through the PVWatts model; to ensure accurate results, our simulation incorporated various technical parameters including the panels’ tilt angles, directional orientation, system efficiency losses, and tracking system dynamics (47). For wind, we utilize NASA’s MERRA-2 dataset for wind speed, direction, pressure, and temperature at various altitudes (48). We employ the Python-based System Advisor Model (PySAM) to simulate wind power generation (49). The wind farm model simulates a detailed layout of 8 rows by 4, totaling 32 turbines, and accounts for wake effects and turbine interactions. Wind speeds are extrapolated to turbine hub heights, adjusted for local terrain and atmospheric conditions using high spatial resolution data from Global Wind Atlas. While satellite-based data for solar and wind resources can have known limitations and biases (50, 51), the sources used remain the most comprehensive and consistently available datasets for simulating renewable generation across broad spatial scales. From the hourly annual generation profiles, we obtain capacity factors at each plant location, accounting for temporal and spatial variation in resource quality. Hourly profiles are simulated on a uniform 5 km grid of candidate points across the contiguous U.S., such that each plant’s buffer zone contains multiple simulated points. For each plant, whereas solar quality tends to be homogenous within the buffer zone, we select the grid point with the highest annual average wind capacity factor within the buffer zone as the representative site.

### Solar, Wind, and Battery Costs.

To estimate the LCOE of solar and wind near each site, we obtain capital and operating expenditures along with financial parameters from refs. 16 and 52. These can be seen in *SI Appendix*, Table S3. We assume a cost of debt of 7%, cost of equity of 8.5%, a debt ratio of 75%, tax rate of 25%, and a project lifespan of 30 y for a calculated discount rate of 6% for both wind and solar PV projects (16, 52). For installation costs, we apply capital cost multipliers to account for regional variation, using data from the U.S. EIA’s regional cost factors and NREL’s ReEDS model (53). For existing fossil sites, we calculate the LCOE at each site for each year between 2024 and 2030; during this time period, the solar PV LCOE is projected to decline an average of $9/MWh and the wind LCOE is projected to decline an average of $7/MWh across all existing fossil sites in the United States, driven by falling capital costs of renewable technologies. For existing renewable sites, we calculate the LCOE at each site for 2025 and 2030. For the scenario with IRA incentives we include the Investment Tax Credit (ITC) and Production Tax Credit (PTC) as outlined in the IRA of 2022, between which the maximum benefit available was applied. Further we incorporate the IRA incentives’ phase-out schedules and eligibility criteria, including the energy community designation for areas identified using (54, 55) with the LCOE adjusted accordingly. The resulting 2024 and 2030 average LCOE of solar and wind at existing fossil plants can be viewed by state in *SI Appendix*, Fig. S4.

### Siting Assumptions.

We assume that renewable projects can be sited anywhere within the developable area identified by the land-use screen and connected to the existing POI. The 20 × 20 km buffer corresponds to a maximum straight-line distance of approximately 14 km (9 miles) from the POI, constraining the length of any required spur line. Longer transmission lines are generally associated with greater permitting complexity, higher costs, and longer development timelines (56). In practice, spur lines are likely to be relatively short as we presume developers would preferentially utilize suitable land closest to the existing substation, although land ownership and site-specific constraints may influence final project layouts. The capital costs of the renewables include a grid connection cost component that includes the spur line development (52). We also assume that solar and wind are not mutually exclusive land uses: because wind turbines occupy only a small fraction of a project’s land area, solar arrays can be sited between turbines without double-counting developable land within the buffer. Therefore, the total maximum local solar and wind potential corresponds to the upper bound on renewable capacity that could be built within the buffer zone of each existing power plant, after applying the land use screen.

### Fossil Plant Operating Costs.

We develop a detailed dataset of existing fossil power plants in the United States using data from the United States Energy Information Administration (EIA) Forms 860 and 923 for the year 2023 and 2022 (25, 57). This dataset includes information on plant location, capacity, fuel type, operational characteristics, heat rates, and variable operating costs for plants with a total generator nameplate capacity of 1 MW or more. Because fuel type and prime mover may not be homogeneous across all units within the same plant, costs and heat rates were compiled on a unit level. To improve precision—in particular for sparse data, like heat rates and variable operating costs—we incorporate cleaned data from NREL’s Regional Energy Deployment System (ReEDS) database, which provides validated engineering assessments based on actual performance data (53). Fuel price data were collected from multiple EIA sources. For natural gas, we took the recent 7-y average incorporating state-level monthly natural gas prices for electric power (supplemented with monthly state-level citygate prices if not available) (58). As oil & gas steam turbines are either mostly gas-based or dual-use technologies, their fuel costs are similarly based on natural gas prices. For coal, we used the recent 7-y average incorporating quarterly prices based on coal shipments to the electric power sector by plant state (59). The 7-y period from 2017 to 2023 was chosen based on data availability, to capture the recent era of high U.S. oil and gas production, and account for fluctuations associated with the COVID-19 economic downturn and subsequent inflationary period. Ownership structures were established using the Environmental Protection Agency’s Greenhouse Gas Reporting Program (GHGRP), supplemented by EIA Form 860 data (25, 60). The estimated variable cost of each fossil unit is composed of the sum of the fuel cost and the variable operating cost per MWh.

For the sensitivity analysis, we took high and low natural gas prices that were one SD above and below the mean, respectively. Natural gas prices in the base case ranged from $2.41 to $7.54/MMBtu (mean: $4.26/MMBtu), with the low scenario ranging from $1.02 to $6.75/MMBtu (mean: $2.40/MMBtu) and the high scenario from $3.74 to $10.00/MMBtu (mean: $6.11/MMBtu). Similarly for coal, base case prices varied from $1.25 to $8.07/MMBtu (mean: $2.56/MMBtu), with the low scenario ranging from $1.20 to $5.45/MMBtu (mean: $2.15/MMBtu) and the high scenario from $1.31 to $10.70/MMBtu (mean: $2.98/MMBtu).

### Identification of Crossover Locations.

We next compare the projected local LCOE of solar and wind with the estimated variable cost of each fossil unit to identify crossover locations, existing fossil power plant sites where the projected local LCOE of solar or wind is lower than the fossil units’ variable cost. These indicate cost-effective opportunities for replacing fossil generation with renewable energy. This is conducted annually from 2024 through 2030 to capture changes in technology costs and fuel prices over time.

### Actionable RE Integration Potential.

Next, we develop an optimization model to identify the cost-effective sizing of the solar and wind capacity (in MW) at each existing power plant, using the hourly solar and wind resource profiles for the 2019 weather year (the optimization does not allocate individual land parcels). This is performed individually for each existing power plant; for notational clarity, the plant index is omitted from Eqs. **1**–**4**. Each plant’s hourly resource input profile is the profile pair of its representative grid point (*Solar and Wind Generation*). The model objective is formulated as follows. For each existing power plant site:

[1] $$Maximize\sum_{t=1}^{8760}(solargen[t]+windgen[t]),$$

with the following decision variables: installed solar capacity solarcap and installed wind capacity windcap and subject to the following constraints:

- Interconnection Capacity Limit, which ensures that at any given moment, the renewable power injection does not exceed the existing interconnection capacity:
  [2] $$solargen[t]+windgen[t]\leq interconnection_{\max}.$$
  The interconnection capacity refers to the maximum amount of power that can be injected from a generation facility into the transmission grid at the POI, under existing network and interconnection agreement constraints (9). While it may not always be the case, for lack of more granular information, we assume it is equal to the rated capacity of the existing power plant as reported in EIA Form 860 (25) and remains unchanged with the addition of surplus resources. As FERC Order 845 stipulates, the combined injection from the existing and surplus resources cannot exceed the capacity originally allocated to the incumbent facility. Therefore, there is no risk of larger thermal loading on nearby transmission lines as a result of the new surplus resources.
- Curtailment Limit, limiting annual curtailment to 5% of total potential generation to maintain economic viability, reflecting market curtailment rates observed for recently deployed utility-scale solar and wind projects across the ISOs (13, 14).
  [3] $$\sum_{t=1}^{8760}(solarcurt[t]+windcurt[t]\leq 0.05\sum_{t=1}^{8760}(potgen[t]).$$
  We define curtailment as foregone renewable production from the surplus resources due to the finite capacity of the shared POI; it does not include periods when renewable export is reduced because the original plant is utilizing available POI capacity. This constraint applies to combined solar and wind output because both resources share a single POI, making curtailment a property of the combined portfolio at the interconnection point. This curtailment constraint serves as a screen for realistically deployable, economically competitive renewable development.
- Curtailment Calculation, where *solarcf*[*t*] and *windcf*[*t*] are the hourly capacity factors for solar and wind, respectively:
  [4] $$\begin{array}{c} solarcurt\left[t\right]=solarcap\times solarcf\left[t\right]-solargen\left[t\right],\\ \;windcurt[t]=windcap\times windcf[t]-windgen[t]. \end{array}$$
  This way, temporal variations in renewable resource availability are represented endogenously and are therefore not counted as curtailment.

Notably, because we have no way of knowing the existing commitments of fossil power plants relative to the grid—such as ramp rates, must-run contracts, capacity commitments, ancillary service obligations for voltage or frequency support—we do not model these constraints. In practice, the incumbent plant would be required to honor them and they may limit the injection of surplus renewable generation through an existing POI. However, many of these constraints can be coordinated at the system level through unit commitment optimization, increasingly accurate renewable forecasting, or colocated battery storage. Further, natural gas plants routinely ramp to accommodate variable renewable generation and fluctuating grid conditions, whereas coal-fired units—albeit generally less flexible because of higher minimum stable generation and slower ramping capability—represent a relatively small subset of the actionable potential: approximately 106 GW or 18% of the 597 GW by 2030.

For the case of existing renewable plants, we do not include the battery capacity as a decision variable, else the model will maximize interconnection utilization and dramatically oversize the storage system, which would be cost-prohibitive. Instead, we fix the rated capacity of the battery storage system as the installed capacity of the existing renewable plant. Across the United States, 6-h battery systems achieve high capacity credits—approximately 60 to 80% of rated capacity, comparable to gas turbines—without being cost-prohibitive, making this a practical choice for our analysis (26–28). While commercial deployment of 6-h battery systems remains limited in the United States today, other regions of the world with rapidly increasing renewable penetrations, most notably Australia, are increasingly installing 6 to 8 h battery systems (61). We include the 19 GW of renewable plants with 30 GWh of existing hybrid battery storage in the dataset, assuming their storage capacity is expanded to 6-h duration.

Whether or not both solar and wind are optimized at the existing POI depends on the viability of the renewable resource and the scenario. Because wind energy exhibits substantial heterogeneity by location, especially relative to solar PV, not every existing fossil site is economically competitive for wind development. Therefore, we allow wind to only be considered at sites where the average annual capacity factor is above 30%, consistent with projects developed in recent years (14). For sites where the wind capacity factor lies below 30%, we optimize solar capacity alone, as in the solar-only scenario. We then estimate the actionable RE integration at existing fossil plants based on the RE potential that was both technically and economically viable: for each individual generation unit (e.g., a gas or steam turbine) for which crossover has occurred, the refined plant-level technically viable RE is adjusted by the proportion of the unit’s capacity to the plant’s total capacity. This reflects the fact that fossil plants are often composed of multiple fuel types and generation technologies, such as facilities that host both combustion turbines and steam turbines.

The actionable RE integration value is also adjusted to reflect cost-effective siting potential. Specifically, although land not excluded by the land use screening may theoretically be available for development, its acquisition may be costly or difficult for a project developer depending on surrounding land uses, particularly when in proximity to urban areas. To account for this, we employ infrastructure density as a heuristic indicator and exclude solar and wind potential at existing plants where built infrastructure exceeds 30% of the land area of the buffer zone, such as peaker plants situated in urban centers. In addition, we apply regulatory restrictions, removing solar and wind potential at sites where county or municipal ordinances prohibit new wind development.

For existing renewable plants, we also subtract the capacity of the existing facility from the total solar and wind potential to obtain the actionable potential, thereby accounting for renewable capacity already installed at the site. Specifically, we adjust the capacity factors of the simulated solar and wind generation profiles by normalizing them to the observed 2024 capacity factors of existing plants. Because hourly generation data for deployed solar and wind facilities are not available nationwide, this normalization is used to account for efficiency differences between older, deployed technologies and current technological benchmarks.

### Interconnection Cost Savings.

To estimate avoided interconnection costs, we use LBNL’s empirical data of completed U.S. generator interconnection projects, which reports total interconnection and network upgrade costs for projects completed between 2019 and 2023 (62). We use the reported regional averages and multiply these costs by the incremental generation capacity accommodated under each surplus interconnection scenario. Estimates are reported as upper-bound savings, reflecting the assumption that surplus interconnection avoids most interconnection and network upgrade costs associated with new standalone projects. We exclude residual site-specific expenditures (e.g., protection equipment or limited reinforcements) and do not account for additional system-level benefits such as reduced bulk transmission investment or colocation synergies.

## Supplementary Material

Appendix 01 (PDF)

## Data Availability

Key modeling results can be explored at scarcitytosurplus.com (63). All other data are included in the manuscript and/or *SI Appendix*.
