# Supplementary material for: Leveraging existing grid infrastructure for rapid US renewable and storage deployment
Source: Proc Natl Acad Sci U S A. 2026 Oct 19;123(43):e2603919123. doi: 10.1073/pnas.2603919123 (PMC13715766; doi:10.1073/pnas.2603919123)
Supplement: Supplementary file 1 [file pnas.2603919123.sapp.pdf]

Supplementary Materials for

Leveraging Existing Grid Infrastructure for Rapid U.S. Renewable and Storage Deployment

Emilia Chojkiewicz[1], Umed Paliwal[2], Duncan Callaway[1], Amol Phadke[2]

[1]Energy and Resources Group, University of California, Berkeley
[2]Goldman School of Public Policy, University of California, Berkeley
*Corresponding author. Email: umed@berkeley.edu

The PDF file includes:

Supplementary Text
1. Case studies
2. Full list of exclusions

Figs. S1 to S10
Tables S1 to S3
References (*1-9*)

Supplementary Text

1. Case studies

Existing fossil power plants

Earthrise Energy is an "independent power producer… that acquires, owns, and operates legacy fossil fuel assets and repurposes the transmission infrastructure for renewable energy development projects, bringing them online faster than typical greenfield renewable energy projects" [1]. The company manages 1.7 GW of gas-fired generation capacity and is developing over 1.5 GW of solar projects primarily in the MISO and PJM market areas.

One example from Earthrise Energy's portfolio is in Gibson City. The Gibson City Energy Center (GCEC) was acquired by Earthrise Energy in December 2021; it is a 237 MW highly flexible natural gas peaking plant. Earthrise Energy plans to develop two solar farms and connect them to the GCEC. The first stage, GCEC - Solar I will be a 135 MW solar PV farm covering approximately 1,300 acres in the eastern portion of McLean County, within five miles of the natural gas plant in Gibson City as seen in Figure S1. The second stage, GCEC - Solar II will be another 135 MW solar PV farm covering approximately 1,300 acres in the southern portion of Ford County, a few miles northwest of the natural gas plant in Gibson City. The lifetime of both projects is projected to be 30-35 years. GCEC - Solar I alone is projected to generate between $24-27 million in economic benefits, including local property taxes during the lifetime of the project.

Earthrise is also developing similar projects at the Shelby County Energy Center Hub, Tilton Energy Hub, Crete Energy Venture Hub, and Lincoln Generating Clean Energy Hub throughout the state of Illinois. An overview of a few of these projects can be found in Table S1.

Another example is Island Green Power (IGP), a utility-scale solar and battery plant developer. Their completed projects total over 1 GW and span the United Kingdom (UK), Spain, Italy, Australia and New Zealand. In the UK, IGP is developing two notable solar projects that are utilizing the interconnection of former/existing power plants: the Cottam Solar Project and the West Burton Solar Project, a summary of which can be found in Table S2.

The Cottam Solar Project is named after its grid connection point, the existing 400kV National Grid substation at the former Cottam Power Station [2]. The Cottam Power Station was a 2,000 MW coal-fired power plant that was closed in September 2019 after over 5 decades of operation. Also at the site is the Cottam Development Centre, a 445 MW natural gas-fired power plant that was commissioned in 1999. The Cottam Solar Project seeks to leverage the opportunity of spare grid connection capacity (in Europe, "interconnection" typically refers to cross-border transmission infrastructure, rather than the grid connection of power plants) by proposing to add 600 MW of solar capacity. This solar capacity would be distributed over several land parcels in close proximity to the substation, within a ~20 kilometer radius, and cover approximately 2,800 acres in total. These land parcels, which have confirmed agreements with the relevant landowners, were selected after thorough evaluation on the existing use and quality of the land, potential environmental or heritage designations, and visibility of the site from local areas. Cable

corridors near existing roads would connect the generated solar power with the substation. The project will also include a 600 MW battery energy storage system.

Not far from the Cottam Power Station, IGP is also developing the similar, yet slightly smaller, West Burton Solar Project [3]. The project is similarly named after its grid connection point, the existing 400kV National Grid substation at the West Burton Power Station. West Burton A was a 2,000 MW coal-fired power plant that was closed in 2023 after nearly 6 decades of operation. West Burton B is an existing 1,270 MW combined cycle gas turbine (CCGT) power plant that was commissioned in 2013. Also at the site is a 49 MW battery energy storage system that was added in 2018. The West Burton Solar Project would similarly leverage the spare grid connection capacity and add 480 MW of solar PV capacity, within a ~20km radius and covering approximately 2,550 acres in total.

Existing renewable plants

Just like the interconnection of existing and retired fossil power plants can be shared with renewables, so can existing renewables and/or storage share interconnection with additional new renewables and/or storage. In the real-world, this oftentimes takes the form of a solar farm being added to an existing wind farm and its existing interconnection point, or vice versa.

There are several examples of this approach around the world:

- In Germany, project developer MLK is constructing a 73 MW solar park in Georgenthal, in close proximity to an existing wind farm [4]. Through this interconnection sharing, the grid connection is overall used more efficiently. There is complementarity between the renewable resources as well: while solar plants produce electricity only during the day, and produce more in summer, electricity production from wind turbines tends to be higher in winter and can be active both day and night.
- In the Netherlands, this is known as “cable pooling” - the pooling of several generators on a single grid connection. While it is most feasible in situations where a solar park can be installed close to an existing wind park, it can also be applied in an industrial location which has existing connection capacity and can accommodate new generation, offering a quick and cost-effective solution to grid constraints. Although project developers may have concerns about combining technologies, Priogen has found through a feasibility study that - at least in the Netherlands - there are in fact very few days in the year where maximum wind output coincides with maximum solar output. As a result, curtailment is only necessary for approximately 1.5% of hours of the year [5]. However, adding battery storage at the same site would further reduce the need to curtail.
- In Australia, the Australian Renewable Energy Agency (ARENA) commissioned a study in 2016 about the national potential for co-locating solar and wind, including both adding a solar farm to an existing wind farm (what they call a “brownfield project”) and developing a new site for simultaneous wind and solar development (what they call a “greenfield project”). They found that approximately 1-1.5 GW of solar PV could be efficiently integrated into the ~4 GW of existing wind farms in Australia, at that point in time. They also found that co-location of wind and solar in greenfield projects could save approximately 3-13% of CAPEX and 3-16% of OPEX [6].

2. Full list of exclusions

The full list of exclusions incorporated in the land use screening:

- Elevation
- Land cover
- Slope
- National Park
- State Park
- National Monument
- State Wilderness
- Conservation Area
- State Conservation Area
- National Forest
- State Recreation Area
- National Grassland
- State Historic or Cultural Area
- National Public Lands
- National Scenic or Historic Trail
- State Resource Management Area
- National Wildlife Refuge
- Wilderness Area
- Wild and Scenic River
- Wilderness Study Area
- Marine Protected Area
- National Recreation Area
- National Scenic, Botanical or Volcanic Area
- National Lakeshore or Seashore
- Inventoried Roadless Area
- Area of Critical Environmental Concern
- Research Natural Area
- Recreation Management Area
- Resource Management Area
- Watershed Protection Area
- Research or Educational Area
- Historic or Cultural Area
- Mitigation Land or Bank
- Military Land
- Access Area
- Special Designation Area
- Approved or Proclamation Boundary
- Native American Land
- Outer Continental Shelf Areas
- Local Park
- Local Conservation Area
- Local Recreation Area
- Local Historic or Cultural Area
- Local Resource Management Area
- Private Park
- Private Conservation
- Private Recreation or Education
- Private Historic or Cultural
- Private Agricultural
- Private Ranch
- Private Forest Stewardship
- Conservation Easement
- Recreation or Education Easement
- Historic or Cultural Easement
- Agricultural Easement
- Ranch Easement
- Forest Stewardship Easement
- Miscellaneous Easement

Supplementary Figures

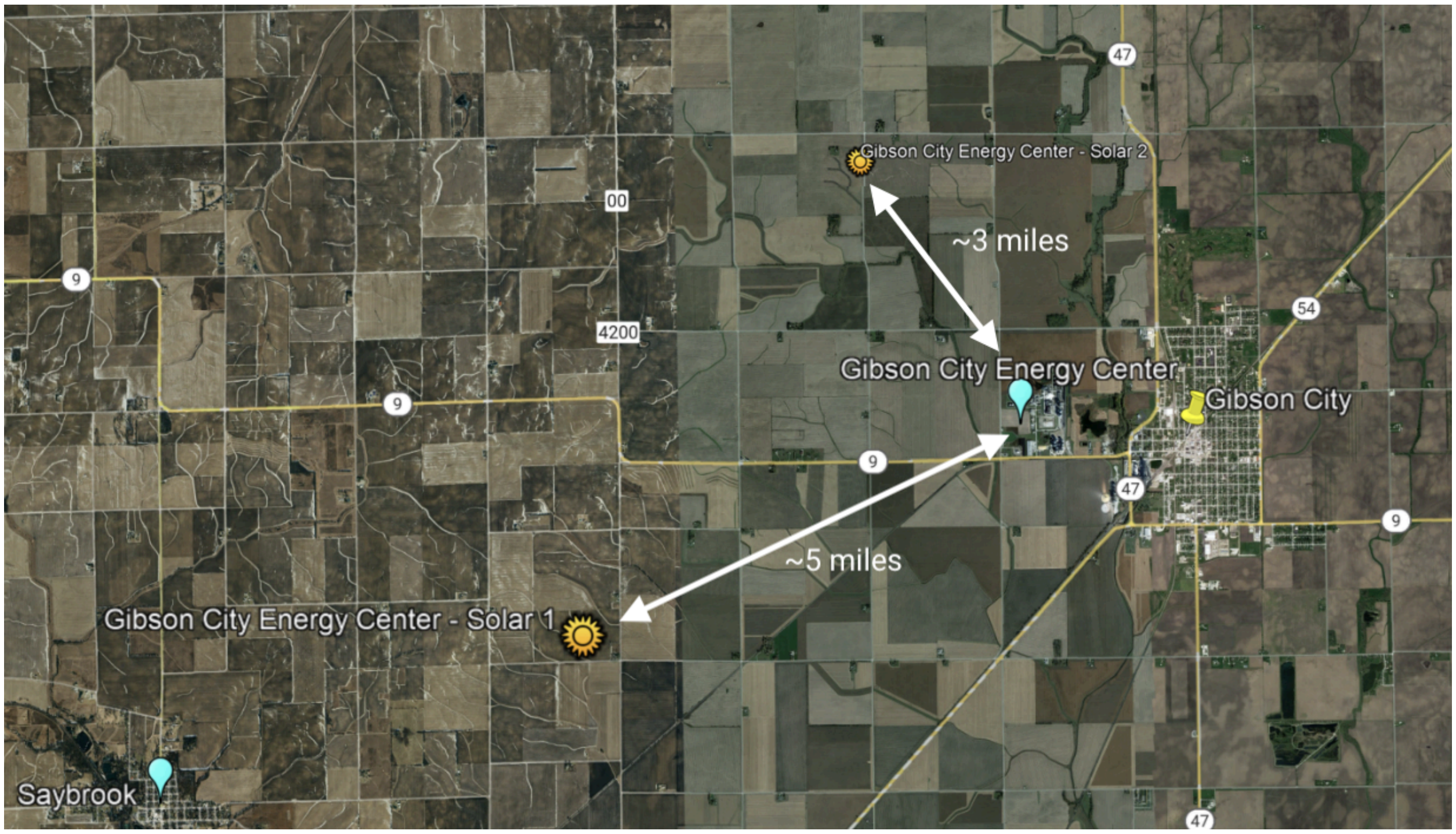

Figure S1. Both GCEC - Solar I and GCEC - Solar II are located within five miles of the gas-fired GCEC.

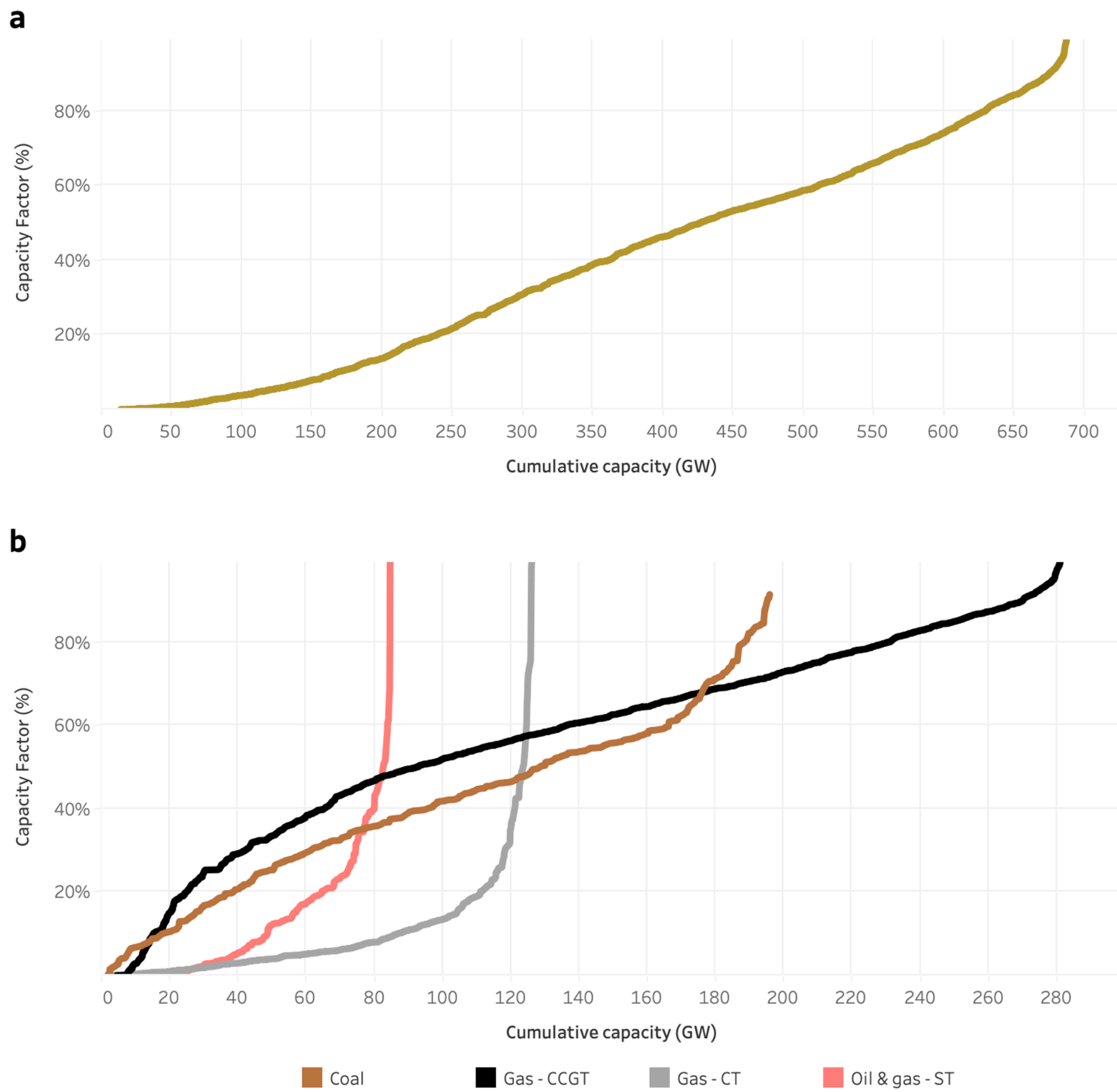

Fig. S2: The capacity factor (representing interconnection utilization) as a function of cumulative capacity across all existing fossil plants in the US (a) and broken down by technology (b). The majority of the installed capacity of gas combustion turbines as well as oil and gas steam turbines operate at less than 20% capacity factor; in total, approximately 250 GW of US fossil capacity operates at a capacity factor of less than 20%. Based on 2023 data [7].

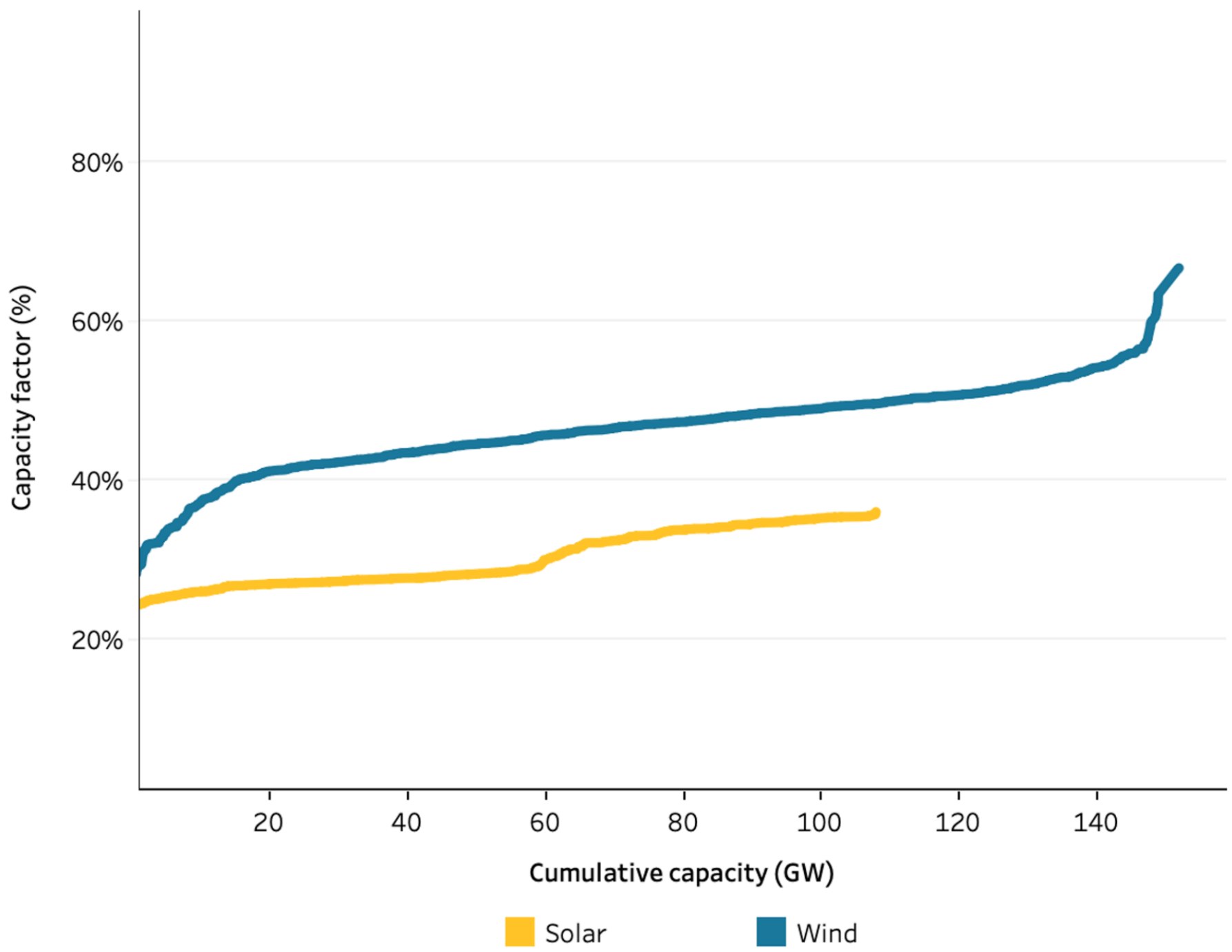

Fig. S3: The simulated capacity factor as a function of cumulative capacity at existing wind and solar plants in the US. The simulated capacity factors are higher than real capacity factors due to differences in efficiency between deployed vintage technologies and the latest technological benchmarks. Based on cumulative capacity as of 2023 [7].

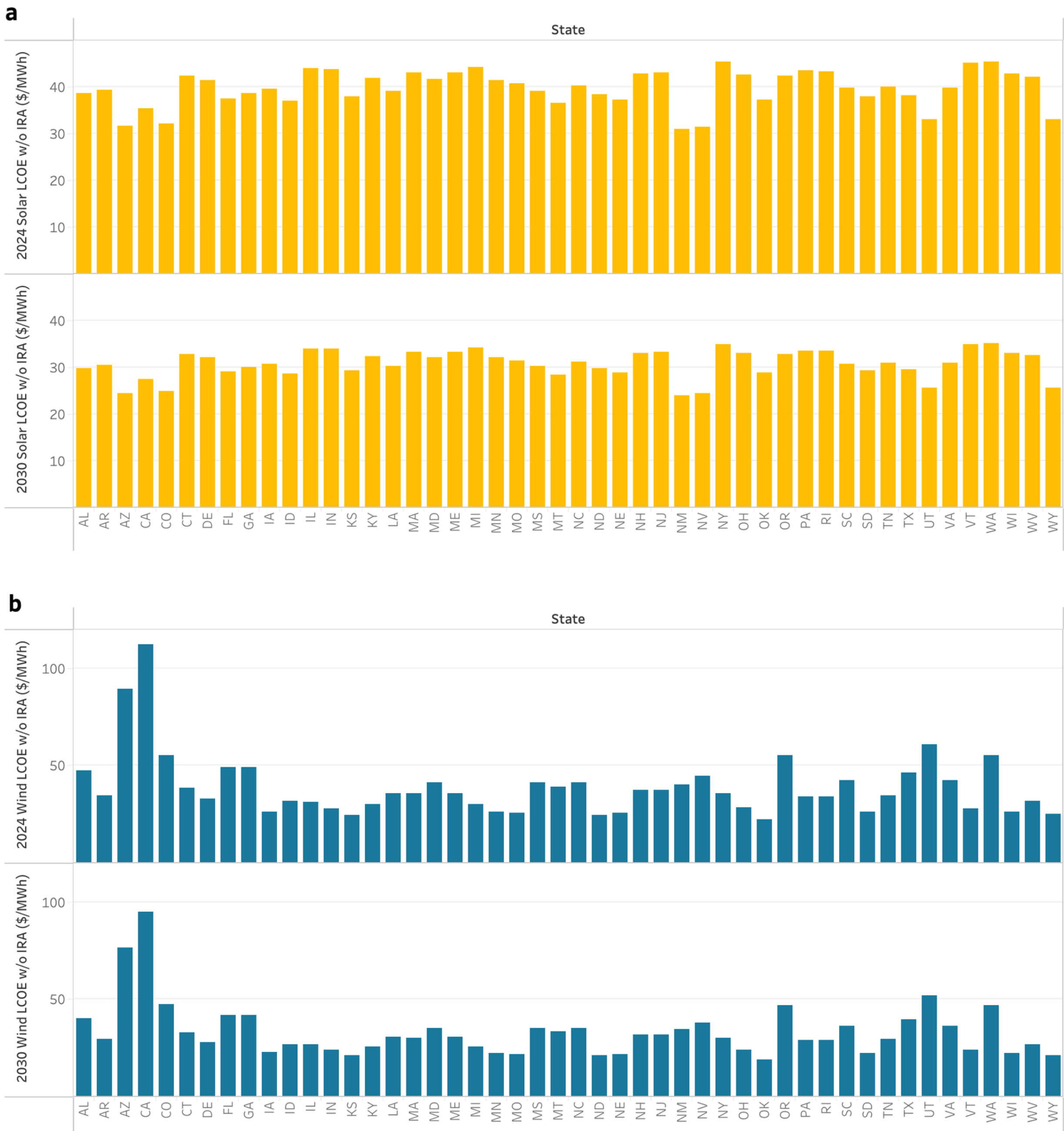

Fig. S4: The calculated 2024 and 2030 average LCOE of solar (a) and wind (b) at existing fossil plants, by state [8,9].

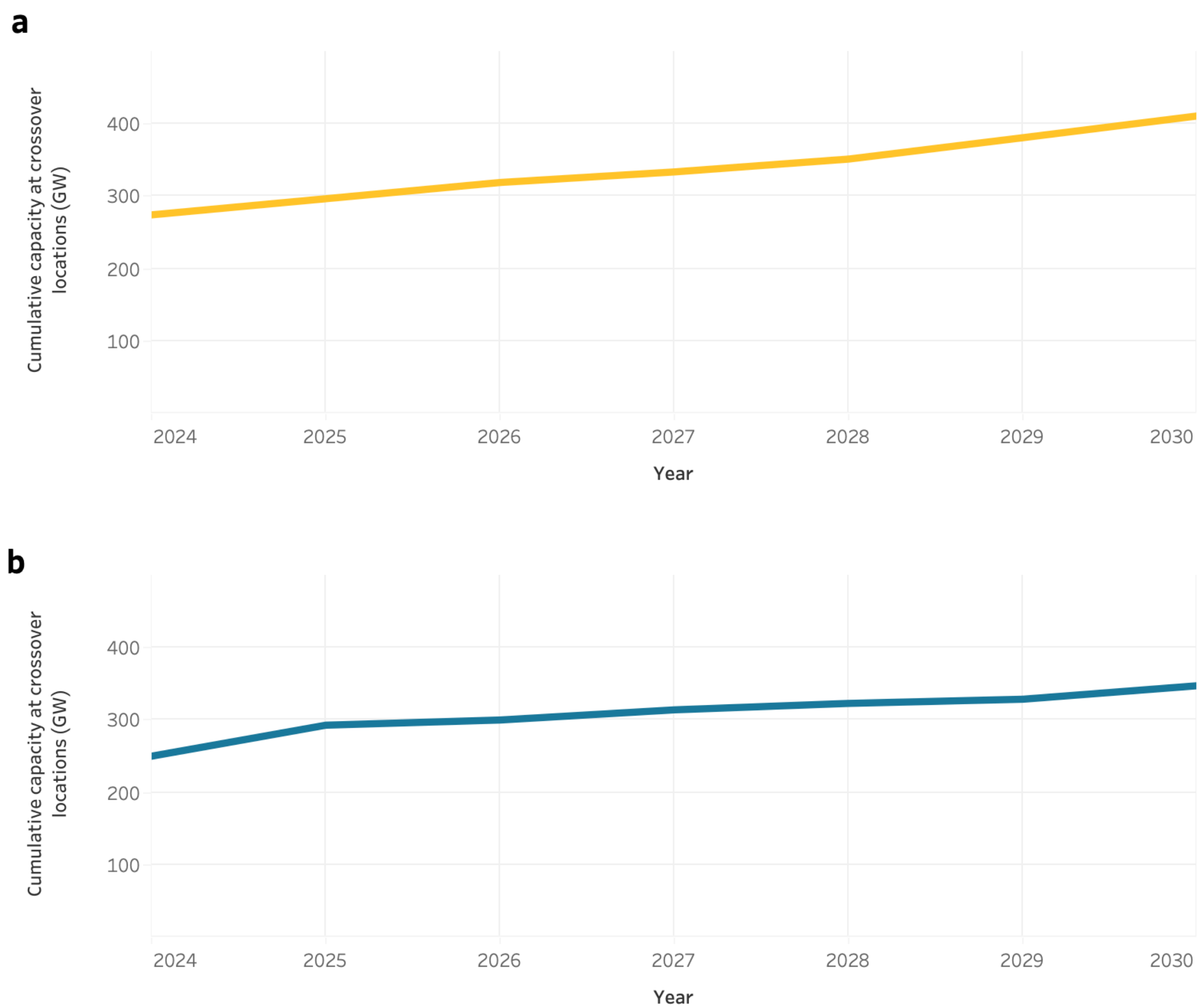

Fig. S5: The cumulative fossil-fired capacity at crossover locations for solar (a) and wind (b), as a function of year. Crossover locations are existing fossil power plant sites where the local LCOE of solar or wind (projected for a given year) is lower than the fossil units' variable cost.

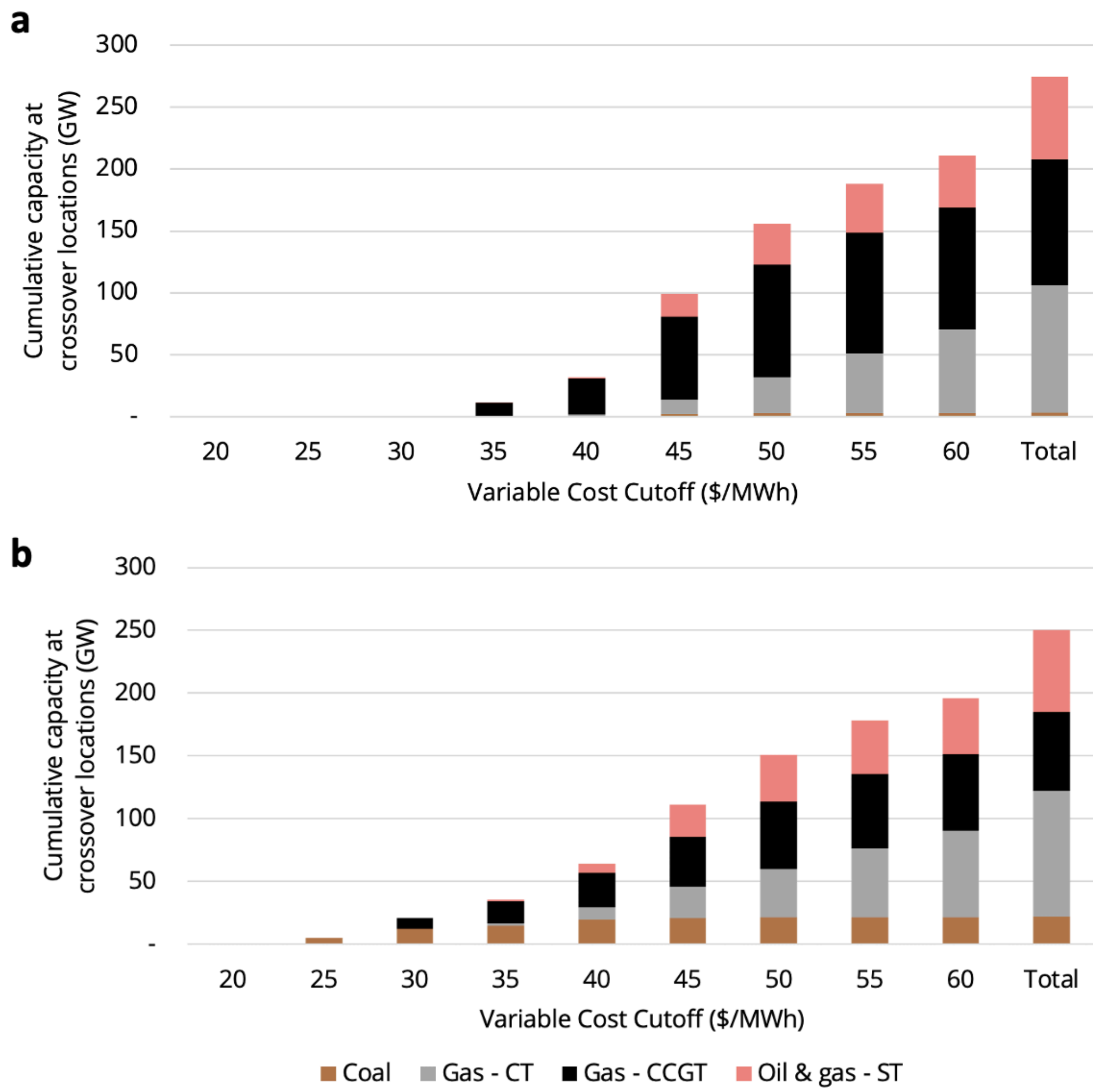

Fig. S6: The cumulative fossil-fired capacity at crossover locations for solar (a) and wind (b) as of 2024, as a function of the variable cost cutoff.

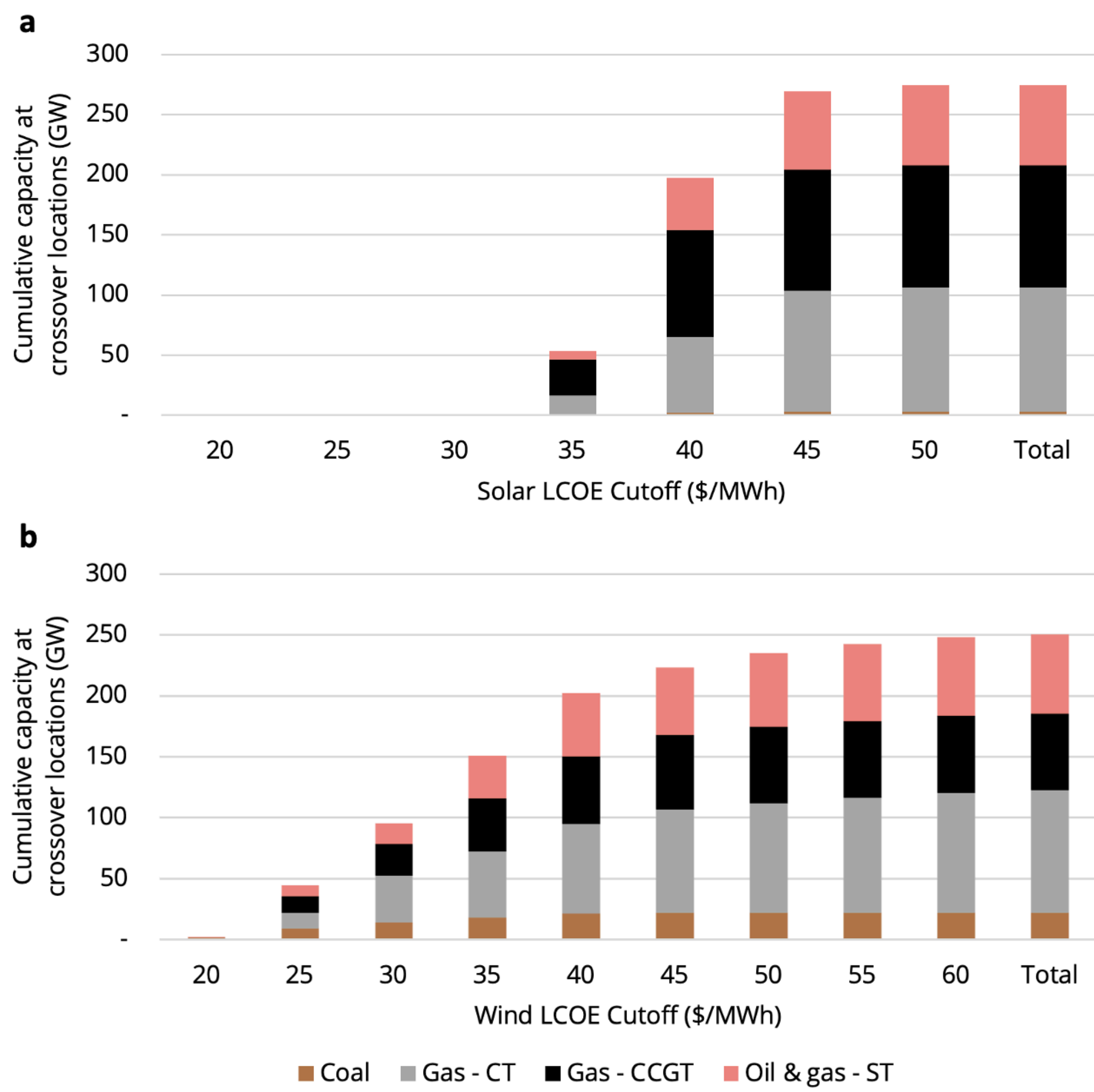

Fig. S7: The cumulative fossil-fired capacity at crossover locations for solar (a) and wind (b) as of 2024, as a function of the local renewable LCOE cutoff.

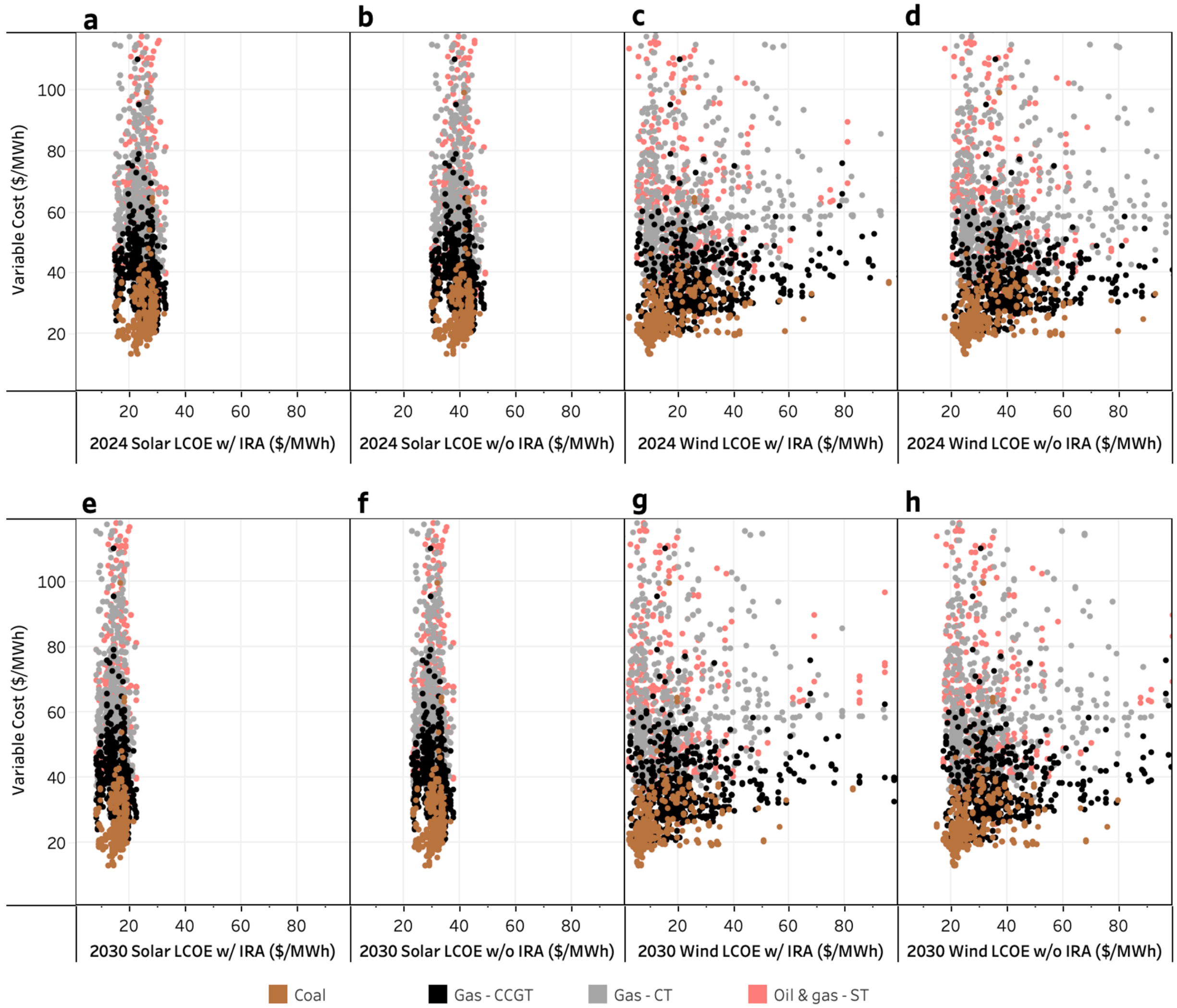

Fig. S8: The LCOE of local solar and wind, with and without IRA incentives (the investment and production tax credits) [8,9], compared to the variable cost (fuel cost plus variable O&M) of existing fossil-fired generating units, both in 2024 (a-d) and 2030 (e-h). Crossover locations are existing fossil power plant sites where the local LCOE of solar or wind (projected for a given year) is lower than the fossil units' variable cost. Each point represents a fossil-fired generating unit, colored by the unit's fuel and technology.

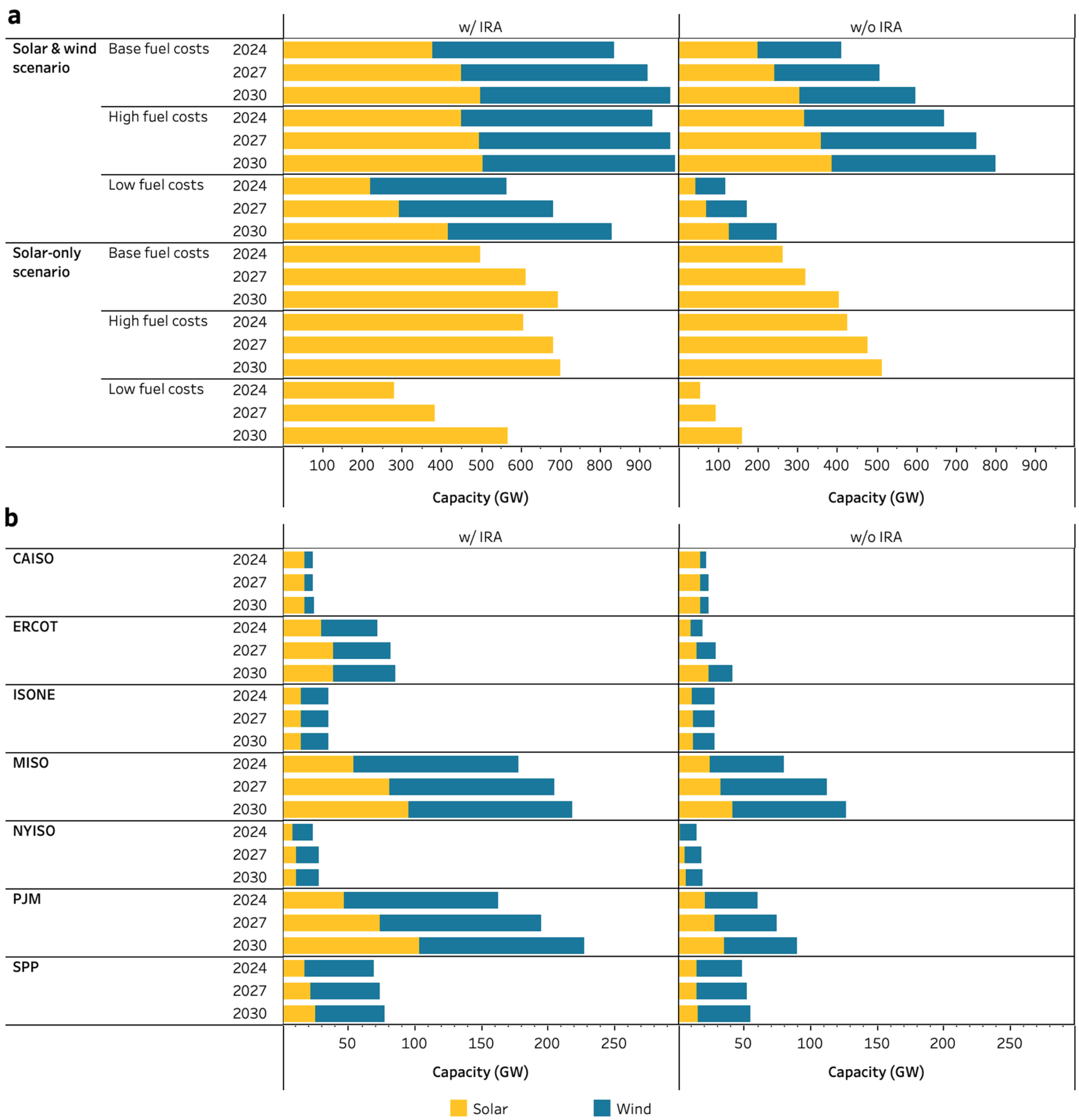

Fig. S9: Actionable renewable integration potential at existing fossil interconnection. (a) The nationwide actionable potential by scenario (solar and wind or solar-only), fuel costs (base, high, low), year (2024, 2027, 2030), and IRA incentives (with or without). **(b)** The actionable potential of solar and wind with base fuel costs by year (2024, 2027, 2030) and IRA incentives (with or without) in each ISO. In the solar & wind scenario, with base fuel costs, IRA incentives (the investment and production tax credits) increase the actionable potential by approximately 40%.

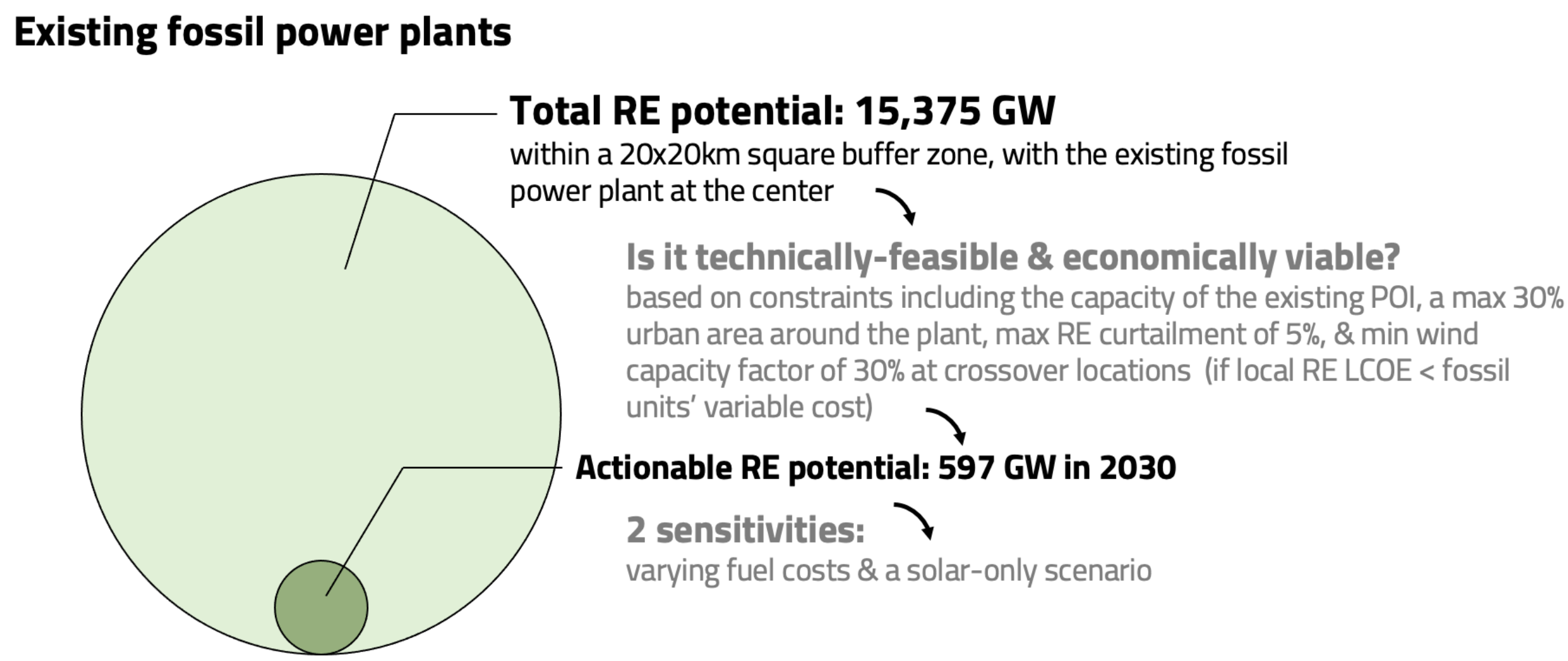

Fig. S10: An overview of the methodology to determine the actionable renewable potential at existing fossil power plants.

Table S1. Summary of domestic case studies.

Existing/former power plant	Existing/former power plant capacity	New power plant	New power plant capacity	Developer	Project Description	Location	Date in-service	Economic impact	Source(s)
Gibson City Energy Center (GCEC) (gas)	237 MW	GCEC - Solar I	135 MW	Earthrise Energy	Earthrise Energy bought the gas plant in 2021 and plans to develop two solar farms nearby. Each solar farm will be sited on ~1,300 acres of land leased from private landowners. Both solar projects will leverage the existing transmission infrastructure at the natural gas plant.	McLean County, Illinois (MISO)	Q1 2026	GCEC - Solar I alone is projected to generate between $24-27 million in economic benefits, including local property taxes during the lifetime of the project (30-35 years).	[1]
		GCEC - Solar II	135 MW			Ford County, Illinois (MISO)			
Shelby County Energy Center (SCEC) (gas)	352 MW	Northwest Solar	150 MW		Earthrise Energy bought the gas plant in 2021 and plans to develop two solar farms nearby. Northwest solar will be sited on ~2,000 acres of land and Glacier Moraine Solar will be sited on ~2,900 acres of land, leased from private landowners, east of the SCEC. Both solar projects will leverage the existing transmission infrastructure at the natural gas plant.	Cumberland County, Illinois (MISO)	Q4 2026		
		Glacier Moraine Solar	210 MW				Q3 2027		
Lincoln Generating Facility (LGF) (gas)	656 MW	Lincoln 1	300 MW		Earthrise Energy acquired the gas plant in September 2022 and plans to develop three solar farms nearby.	Will County, Illinois (PJM)	Q4 2026		
		Lincoln 2	200 MW				Q2 2027		
		Lincoln 3	100 MW				Q3 2027		

Table S2. Summary of international case studies.

Existing/former power plant	Existing/former power plant capacity	New power plant	New power plant capacity	Developer	Project Description	Location	Date in-service	Economic impact	Source(s)
Cottam Power Station	2,000 MW decommissioned coal-fired capacity; 445 MW of existing gas-fired capacity	Cottam Solar Project	600 MW	Island Green Power	Sites for these solar projects were chosen in order to utilize the existing grid connection infrastructure left behind by retired coal-fired power plants. The project will also include a 600 MW battery energy storage system.	Retford, United Kingdom	Late 2020s		[2]
West Burton Power Station	2,000 MW decommissioned coal-fired capacity; 1,270 MW of existing gas-fired capacity	West Burton Solar Project	480 MW			Retford, United Kingdom			[3]

Table S3. Solar PV and wind cost assumptions [8,9].

Year	Solar PV capital cost ($/kW)	Solar PV fixed O&M ($/kW-yr)	Onshore wind capital cost ($/kW)	Onshore wind fixed O&M ($/kW-yr)
2024	1026.10	17.06	1695.00	38.69
2025	1056.07	15.56	1703.01	39.04
2026	956.69	16.47	1685.91	38.30
2027	893.95	16.43	1647.51	37.27
2028	851.78	16.08	1627.63	36.73
2029	804.77	15.78	1607.55	36.17
2030	779.58	15.50	1587.06	35.66

References

[1] “Who We Are”. Earthrise Energy, 2025. https://earthriseenergy.com/

[2] “The Project”. Cottam Solar Project, 2025. https://www.cottamsolar.co.uk/

[3] “The Project”. West Burton Solar Project, 2025. https://www.westburtonsolar.co.uk/

[4] R. Heinen. “MLK builds 73 MW solar park and couples solar with wind”. Energate, 2024. https://www.energate-messenger.com/news/244359/mlk-builds-73-mw-solar-park-and-couples-solar-with-wind

[5] “Cable Pooling for Solar”. Priogen, 2024. https://priogen.com/cable-pooling-for-solar/

[6] “Co-location Investigation: A study into the potential for co-locating wind and solar farms in Australia". Australian Renewable Energy Agency (ARENA), 2016.

[7] “Form EIA-923 detailed data with previous form data (EIA-906/920)”. Energy Information Administration (EIA), 2024. https://www.eia.gov/electricity/data/eia923

[8] “LCOE Data Viewer”. Bloomberg New Energy Finance (BNEF), 2024.

[9] (NREL, 2024). “Annual Technology Baseline 2024”. National Renewable Energy Laboratory (NREL), 2024. https://atb.nrel.gov/electricity/2024/data
